# The Minoan Thera eruption predates Pharaoh Ahmose: Radiocarbon dating of Egyptian 17^th^ to early 18^th^ Dynasty museum objects

**DOI:** 10.1371/journal.pone.0330702

**Published:** 2025-09-10

**Authors:** Hendrik J. Bruins, Johannes van der Plicht

**Affiliations:** 1 Ben-Gurion University of the Negev, Jacob Blaustein Institutes for Desert Research, Swiss Institute for Dryland Environmental and Energy Research (SIDEER), Midreshet Ben-Gurion, Israel; 2 University of Groningen, Centre for Isotope Research, Groningen, the Netherlands; Austrian Academy of Sciences, AUSTRIA

## Abstract

The huge volcanic eruption at Thera (Santorini), situated in the Aegean Sea, occurred within the Late Minoan IA archaeological period. However, its temporal association with Egyptian history has long been a controversial subject. Traditionally, the eruption was placed in the early 18^th^ Dynasty, associated with Pharaoh Thutmose III as the youngest option or with Pharaoh Nebpehtire Ahmose as the oldest possibility. We investigated museum objects from the 17^th^ and early 18^th^ Dynasty, at the transition from the Second Intermediate Period to the New Kingdom, a period hardly studied with radiocarbon dating. Our research facilitated the first-ever direct radiocarbon time comparison between this Dynastic transition period and the Minoan Thera eruption. Detailed results are presented of a mudbrick from the Ahmose Temple at Abydos (British Museum), a linen burial cloth associated with Satdjehuty (British Museum), and wooden stick shabtis from Thebes (Petrie Museum), evaluated within a comprehensive context of historical Egyptian chronology options. Since the above items cannot be arranged in a stratigraphic sequence, Bayesian analysis could not be used. We adopted an alternative strategy within radiocarbon time space. Comparing our uncalibrated dates of 17^th^ and early 18^th^ Dynasty objects with a robust series of uncalibrated radiocarbon dates for the Minoan Thera eruption, it becomes clear that the two data sets have a different time signature. The Minoan eruption is **older** than the reign of Nebpehtire Ahmose, the first king of the 18^th^ Dynasty, who reunited Upper and Lower Egypt. Our calibrated results support a **low** chronology for his reign and the beginning of the New Kingdom. Previous radiocarbon dates of king Senusert III support a **high** chronology for the Middle Kingdom. Therefore, the Second Intermediate Period, sandwiched in between these united Egyptian Kingdoms, embodies a significant time interval, as also indicated by Bennett’s genealogical studies of the El-Kab governors.

## Introduction

### The Thera eruption during the Late Minoan IA period

**Fig 1:**
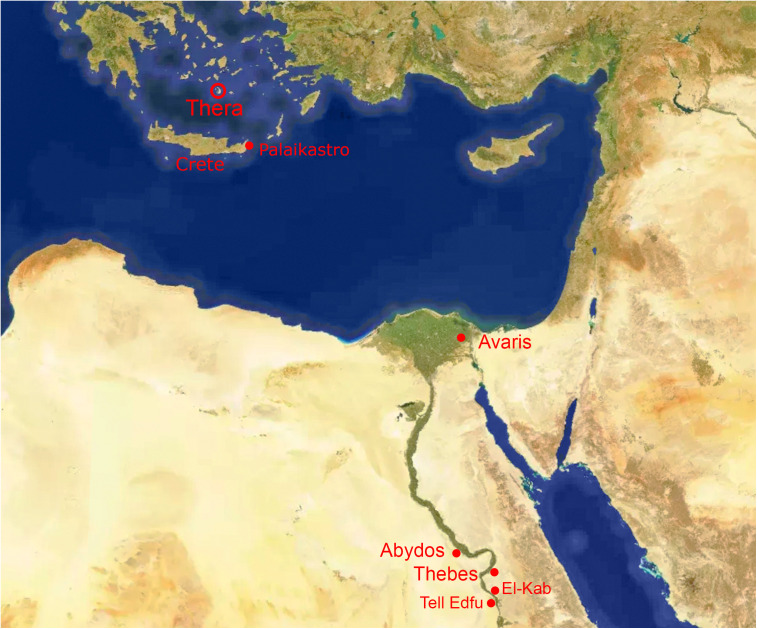
The eastern Mediterranean region and Egypt, showing the location of the Thera (Santorini) volcano and other places mentioned in the text. Based on Mapcarta, the open map with CC BY license © OpenStreetMap, Mapbox, and Mapcarta.

The Santorini or Thera volcano is situated in the Aegean Sea (Greece), about 120 km north of Crete (Fig 1). Its caldera is surrounded by the small islands of Thera, Therasia and Aspronisi. The volcano has produced quite a number of major explosive eruptions during the Quaternary [1]. The most famous one occurred during the Late Minoan IA, an archaeological period traditionally dated from about 1600/1580–1480 BCE [2,3]. Next to the volcano, the Minoan eruption buried the town of Akrotiri in southern Thera under thick layers of tephra [4]. Fine tephra was also found in eastern Crete, transported through the atmosphere by south-easterly winds [5–10].

Farther away, the Minoan eruption caused widespread deposition of volcanic tephra in the eastern Mediterranean region, in deep-sea sediments [11–14], Rhodes [15], Turkey [16], and possibly the Nile delta [17]. In addition, pieces of pebble-size pumice from the Minoan eruption, floating in the water, were transported by Mediterranean sea currents to shores around the eastern Mediterranean, including Egypt [18,19] and Sinai [20].

The erupted volume of the Minoan Thera eruption has been revised upward from ca 60 km^3^ [21] to about 80 km^3^ dense-rock equivalent (DRE), which would make it the largest known volcanic eruption in the world during the Holocene [22]. For comparison, the famous and well-documented eruption in 1883 of the Krakatau volcano [23–25], situated in the Sunda Strait between Java and Sumatra, produced significantly less magma, ca 13 km^3^ DRE. Nevertheless, the Krakatau eruption caused devastating tsunamis that killed at least 36,000 people and destroyed about 300 villages [26]. Dwelling upon the effects of the Krakatau eruption, the Greek archaeologist Marinatos [27] advanced a hypothesis suggesting that coastal settlements in Minoan Crete may also have been hit by tsunamis caused by the Thera eruption.

Conclusive evidence to substantiate this hypothesis remained elusive for a long time [15,28]. However, following initial archaeological indications at the coastal Minoan town of Palaikastro in north-eastern Crete [3,29], widespread geoarchaeological tsunami deposits, mixed with fine volcanic tephra from the Minoan Thera eruption, were discovered by the first author along the coast of Palaikastro and investigated together with his colleagues [8,30]. Subsequently, more tsunami deposits related to the Minoan eruption were found along the Mediterranean coast, near Caesarea in Israel [31], at the Minoan town of Malia in Crete [32], and in Turkey [33]. The recognition of palaeo-tsunami signatures requires expertise in the earth sciences [34].

### Timing the Minoan Thera eruption in relation to Dynastic Egypt

Time is a critical dimension in studies dealing with the past, whether from a historical, archaeological or geological perspective. Different dating systems developed in each of these disciplines, leading *sometimes* to a problematic comparison of “time oranges” with “time apples”. Egyptian historical chronology has long been a basic framework in the eastern Mediterranean region to “calibrate” relative archaeological periodization into “real time”. The Minoan Thera eruption has traditionally been associated with the 18^th^ Dynasty around 1500 BCE [3,4,15,18,27,35–38]. Suggestions have been made to link the time of the eruption with specific pharaohs, including Hatshepsut/Thutmose III [37] and Nebpehtire Ahmose [39].

Each dating method has its own history, development, and potential time resolution. Historical dating, based on written sources, as well as archaeological dating, based on material cultural data, preceded the development of time measurements based on natural science. Radiocarbon dating is a relative newcomer, based on nuclear physics [40]. Though ^14^C dating has its own limitations, it provides an independent measurement of time, intrinsically unrelated to the interpretation of literary data, ceramic sequences, and their interconnections. Radiocarbon dating of suitable organic materials in Egyptian, Aegean and Near Eastern archaeological, historical and geological contexts is a necessary approach to apply the same methodology across the region and across disciplines [41,42], as well as to evaluate Egyptian historical chronologies [43].

### Radiocarbon dating of the Minoan Thera eruption: Changes in calibration curves and dates

Standard deviations of radiocarbon dates of the Minoan eruption were rather large during the 1980s. Their calibration into calendar years, based on ^14^C measurements of dendrochronological datasets then available, indicated an eruption most likely in the Second Intermediate Period before the 18^th^ Dynasty [44–47]. A number of archaeologists became convinced to accept a higher chronology, also on the basis of alternative interpretations of material cultural interconnections [48–52]. One of the first to explicitly place the Theran eruption within the Second Intermediate Period was Betancourt in 1987 [44]: “*In conclusion, if we were to ignore earlier prejudices completely and erect a new Aegean chronology today, it would be somewhat different from the received tradition. This author withdraws many of the opinions he expressed a decade ago (Betancourt and Weinstein 1976)* [53]*; the Aegean Late Bronze Age probably began during the Hyksos period, and radiocarbon was correct all along*”. Also Christos Doumas, the chief excavator at Akrotiri since the death of Marinatos in 1974, changed his initial understanding and accepted a high chronology [54,55].

The calibration curve, linking radiocarbon time to calendar time, has not remained static, as more detailed ^14^C measurements of dendrochronological datasets became available over the years. When the IntCal98 calibration curve [56], released in 1998, was in use, the Minoan Thera eruption was dated quite early in the 17^th^ century BCE by Bayesian analyses, to ca 1650–1620 BCE [54], and to 1663–1599 BCE [57]. Since IntCal98 [56], hundreds of additional dendrochronological ^14^C dates have been measured with high precision [58], which have become incorporated in the present IntCal20 calibration curve [59], available since 2020. The new calibration curve also affected the possible age of the Minoan Thera eruption [58]. Consequently, new Bayesian analyses concerning the time of the Thera eruption were conducted by Manning [60], using the latest IntCal20 calibration curve, which resulted in the following 1σ and 2σ age ranges: 1606–1589 BCE (68.3% probability), 1609–1560 BCE (95.4% probability). Indeed, compared with his Bayesian calibration results [54] based on IntCal98 [56], the age range for the Thera eruption has become younger by roughly 40 years [60], using Bayesian analyses with the updated calibration curve IntCal20 [59].

Nevertheless, Manning maintained that the volcanic event occurred during the Second Intermediate Period [60]. However, Pearson et al. [61] suggested a younger date in the 16^th^ century BCE for the Thera eruption and possible association with the reign of Ahmose, based on their annual dendrochronological datasets for the period 1700–1500 BCE. Another basis for their proposition is related to the Tempest Stela erected by Ahmose, describing a severe catastrophic rainstorm, interpreted by Ritner and Moeller [62] as having been caused by the Thera eruption. Thus, Pearson et al. [61] suggested that the volcanic event was probably coeval with the reign of Ahmose.

Concerning the identity of pharaoh Ahmose, a brief elucidation is required here in the context of our article. The 17^th^ Dynasty king Senakhtenre was until recently only known with his throne name (prenomen), i.e., Senakhtenre. He was the grandfather of king Nebpehtire Ahmose [63], founder of the 18^th^ Dynasty. Possible birthnames (nomen) were tentatively suggested, Tao [64] or Siamun [63]. However, in 2012, Biston-Moulin [65] discovered at Karnak (Thebes) that Ahmose is the real birthname (nomen) of king Senakhtenre. Therefore, he is in fact Ahmose I, a term usually given to his grandson, who can now be regarded as Ahmose II. But the 26^th^ Dynasty king Amasis is often referred to as Ahmose II. To avoid confusion, Cahail [66] recommended using both the throne name and birthname for the respective Ahmose kings of the 17^th^ to 18^th^ Dynasty, and/or using the Roman numerals (I) and (II) in parenthesis. The Pharaoh Ahmose in our article is Nebpehtire Ahmose (II), even if only the name Ahmose is used for brevity.

Manning [67] discussed and evaluated the vexing problems of dating spread on the radiocarbon calibration curve plateau 1620–1540 BCE in relation to the Minoan Thera eruption. He also addressed the radiocarbon dates of the olive shrub at Therasia and came to different conclusions than Pearson et al [68]. His latest Bayesian analyses, published in 2024, concerning the date of the Minoan Thera eruption resulted in the following age ranges: (1σ) 1612–1602 or 1613–1602 BCE, (2σ) 1616–1589 or 1618–1584 BCE. The peak value with the highest probability of his modeled eruption date is situated around 1608 BCE [67].

### Radiocarbon dating of Dynastic Egypt

Concerning ancient Egypt, a large radiocarbon study was conducted between 1984 and 1995, focusing on monumental buildings, including pyramids and tombs of the Early Dynastic Period, the Old and Middle Kingdom [69,70]. The authors sampled organic material from monuments linked to specific kings or sections of Dynastic history. They dated charcoal, wood, plant remains, and humates from mudbricks and mud mortar in between building stones. The precision of radiocarbon measurement was often limited in those days, leading to relatively large standard deviations. Moreover, results from the same monument were often inconsistent, whilst their combined average tended to produce older dates than historical age assessments for the Old Kingdom [70]. A reanalysis of the above radiocarbon dates regarding 4^th^ Dynasty monuments was conducted by Dee et al. [71], using the OxCal calibration program [72] and its function to detect outliers [73]. Their Bayesian analysis and removal of outliers produced new radiocarbon calibration results, showing much closer agreement with historical Dynastic age assessments [71].

The most comprehensive and robust radiocarbon investigation so far of Egyptian Dynastic history was published by Bronk Ramsey et al. [74], including the Old, Middle and New Kingdoms. Their investigation was based on short-lived plant remains, usually from museum collections, associated with specific pharaohs or sections of the historical chronology. A number of samples from the monumental buildings project [69,70] were also used in their ^14^C measurements. Bayesian models were developed by the authors [74], combining their radiocarbon dates with historical data of the sequence and reign-lengths of the successive pharaohs in order to model the accession year of each pharaoh in the Old, Middle and New Kingdoms. The authors emphasized that “*the radiocarbon dates provide the only linkage in the model to the calendar time scale*” [74]. Their Bayesian model tends to favor a higher (older) historical Egyptian chronology [75] rather than a lower (younger) chronology [76].

However, the historically problematic First and Second Intermediate Periods were not included in their investigation for obvious reasons. Bayesian sequence modelling, the principal methodology in their research, cannot be conducted for these Intermediate Periods. Knowledge about sequences of kings and their respective reign lengths in these periods are usually uncertain and full of lacunas, but such information is essential to enable sequence modelling [77]. Moreover, it is difficult to find organic samples in museums linked to specific historical figures of the First and Second Intermediate Periods.

### Objectives of our investigation

We aimed in our research to obtain samples of Egyptian museum objects associated with the transition phase from the 17^th^ to early 18^th^ Dynasty in order to get actual radiocarbon measurements of this important historical period, which were lacking so far. Thus, we can directly compare ^14^C dates of Egypt’s transition from the Second Intermediate Period to the New Kingdom with ^14^C dates of the Minoan Thera eruption.

The temporal position of Pharaoh Nebpehtire Ahmose forms a key anchor regarding these research objectives. The reign of Ahmose began as king of the 17^th^ Dynasty in Upper Egypt, dominated by the ancient cities of Thebes and Abydos (Fig 1). His reign lasted 25 years and 4 months, according to Manetho [78]. Politically, the beginning of the New Kingdom may be placed at the conquest of Avaris (Fig 1) when king Ahmose defeated the Hyksos empire and reunified Upper and Lower Egypt. The conquest of Avaris occurred not later than ca year 18 in the reign of Ahmose [79]. However, in terms of traditional historical classification following Manetho, the beginning of the 18^th^ Dynasty is placed with the accession year of Ahmose, for which, unfortunately, no historical dates have so far been found [80].

Ahmose erected the so-called Tempest or Storm Stela early in his reign at Karnak, located in the northern part of Thebes (Fig 1). The text of this remarkable stela describes an extraordinary severe rainstorm, characterized by clouded skies and darkness, which caused widespread destructions, apparently witnessed by Ahmose himself. Some of the phenomena mentioned in the stela were interpreted by a number of scholars as possibly related to the volcanic eruption at Thera [62,81–84]. Other scholars have argued against the above linkage [85–87], suggesting alternative translations and interpretations of the hieroglyphic text. Therefore, the important question is whether the reign of Nebpehtire Ahmose is coeval with the Minoan Thera eruption?

Historically, the time period of Ahmose’s reign is by no means fixed. Egyptological age assessments of his rule range from 1580–1557 BCE [88] to 1524–1499 BCE [89]. Using Bayesian sequence analysis of a series of 18^th^ Dynasty radiocarbon dates, coupled with historical information, Bronk Ramsey et al. [43] modeled the accession year of Ahmose. The resulting age ranges are 1566–1552 BCE (1σ) and 1570–1544 BCE (2σ), using OxCal [72,90] with the IntCal04 calibration curve [91]. A different Bayesian model by Quiles et al [92], focusing on the 18^th^ Dynasty, included astronomical Sothic and Lunar data, historical reign length options, and radiocarbon dates of Sennefer’s tomb and the eastern cemetery at Deir el-Medineh. Using the IntCal09 calibration curve [93] and OxCal [72,90], this model yielded younger age ranges for the accession year of Ahmose: 1557–1537 BCE (1σ) and 1564–1528 BCE (2σ) [92].

However, both Bayesian models did not include radiocarbon dates specifically related to Nebpehtire Ahmose or the other early kings of the 18^th^ Dynasty prior to Thutmose III. This shortcoming was acknowledged by Bronk Ramsey et al. [43]:: “*there are no dates for specific reigns before that of Thutmose III, and so dates earlier than this are based primarily on the reign-length information included in the model*”. The preceding Second Intermediate Period was not involved in these investigations. Therefore, the modeled age ranges for the accession year of Ahmose should be considered tentative.

Our investigation focused on this lack of radiocarbon dates for Egypt’s 17^th^ Dynasty and early 18^th^ Dynasty. Thus, we selected museum objects associated with this transition period suitable for radiocarbon dating. Our results enabled the first-ever chronological comparison of this Egyptian historical phase with the Minoan Thera eruption, ***using the same methodology,*** ^***14***^***C, to measure time***. We achieved clear results, showing that the two groups of dates, i.e. Egypt’s 17^th^ to 18^th^ Dynasty transition period and the Minoan Thera eruption are ***not synchronous***. Moreover, the radiocarbon dating results of the individual museum objects provide novel chronological information, relevant in terms of Egyptology.

## Materials and methods

The selection of organic objects in museums, linked to the transition period of the 17^th^ to early 18^th^ Dynasty, proved to be difficult, because not much material related to historical figures of this period is available in general. In addition, not all museums approached were willing to cooperate, as small fragments have to be extracted from the organic objects, i.e., destructive sampling, to facilitate radiocarbon dating. However, the British Museum (London) and the Petrie Museum of Egyptian and Sudanese Archaeology (University College London) kindly gave their permission regarding a number of requested objects related to the transition period from the Second Intermediate Period to the New Kingdom. The selected museum objects are necessarily evaluated in detail with regard to their acquisition and archaeo-historical context, which is essential for age assessment in relation to our radiocarbon dating results.

Additional information regarding the ethical, cultural, and scientific considerations specific to inclusivity in global research is included in the Supporting information (SX Checklist).

The most important object in our investigation is a mudbrick stamped with the throne name Nebpehtire of pharaoh Ahmose, kept in the British Museum. Another relevant item, also from the British Museum, is a linen burial cloth associated with Queen Satdjehuty. She was the second wife of the 17^th^ Dynasty Pharaoh Seqenenre Tao. The latter king was succeeded by Kamose, who was the predecessor of Nebpehtire Ahmose [63]. Another series of samples associated with the 17^th^ Dynasty are wooden stick shabtis, originating from Thebes (Fig 1), which were collected by Sir Flinders Petrie and are kept in the Petrie Museum [94,95]. We received samples from six stick shabtis.

Concerning the Ahmose mudbrick, the methodology to measure the time of mudbrick production is based on ^14^C dating of plant (straw) fragments, which were added to soil mud from Nile sediment in the process of mudbrick fabrication [96]. However, the alluvial Nile soil may also contain older inherited plant remains and organic compounds, transported by the Nile or from past human activities in the soil, which could result in radiocarbon dates older than the actual time of mudbrick production [70,97]. Studying mudbrick morphology at the microscopic scale may provide additional information regarding organic constituents. Therefore, an already detached but intact aggregate (lump) of the Ahmose mudbrick, having a size of a few centimeters in length, width and thickness, was sent to a specialized laboratory [98] for impregnation with polyester resin under vacuum to harden the soft mudbrick. A thin section was made for microscopic examination.

Radiocarbon dating of all samples was carried out at the Centre for Isotope Research of Groningen University, the Netherlands. The samples with GrA number were measured with the 2.5MV Tandetron AMS [99], which was replaced in 2017 by a Micadas AMS system [100]. The dates measured by the latter system have a GrM number. Regarding quality control, the Groningen radiocarbon laboratory always participates in international ^14^C intercomparisons, including the recent Glasgow International Radiocarbon Intercomparison (GIRI), see Scott et al. [101]. In addition, extensive ^14^C intercomparison dating was conducted between selected laboratories, including Groningen, using large series of dendrochronologically dated samples [102,103]. The results underline the reliability and excellent quality of the Groningen radiocarbon laboratory. Concerning the time period of the Minoan Santorini eruption, tree rings of a new series of dendrochronologically dated oak wood from the Netherlands were ^14^C dated in Groningen [104] and incorporated into the IntCal20 calibration curve [59].

Pretreatment of our samples usually involved the standard acid-base-acid (ABA) procedure, also termed acid-alkali-acid (AAA). But samples with low amounts of carbon were only pretreated with acid. Following pretreatment, the carbon in each sample was combusted to CO_2_ gas, subsequently reduced to graphite [105]. Next, the graphite was pressed into targets mounted in a sample wheel, which was loaded into the ion source of the AMS machine for measurement of ^12^C, ^13^C, and ^14^C amounts. For extremely small samples the graphite procedure is not used, but the CO_2_ gas source of the combusted sample is used to measure ^14^C.

The radiocarbon dates are reported by convention in ^14^C years BP [106], using the oxalic standard and Libby half-life, and including normalization for isotope fractionation. The ^14^C dates are calibrated from radiocarbon years into calendar years, using the the OxCal software program v4.4.4 [72,90] and the IntCal20 calibration curve [59], which is based on dendrochronological tree ring dates covering the past 14,000 years. The cal prefix – cal BCE in our study – specifies that the dates result from radiocarbon dating and subsequent calibration.

The ^14^C measurement is mathematically represented by a probability distribution called Gaussian. The central (median) value of this distribution, having the highest probability, is reported in ^14^C years BP ± the uncertainty value, known as the standard deviation (σ). The uncertainty factor may relate for example to the quality of the sample, pretreatment procedures and AMS operational stability. For a Gaussian function, the 1σ number corresponds to 68.3% probability and 2σ to 94.5%.

Calibration of the Gaussian BP date into calendar years results in a probability distribution with an irregular shape, which is no longer Gaussian. This is caused by the non-linear, irregular shape of the calibration curve, resulting from variations in the ^14^C content of the atmosphere through time. Hence the relationship between ^14^C time (BP) and calendar time (cal CE or BCE) is not linear. The 1σ and 2σ probabilities of a calibrated non-Gaussian probability distribution are calculated by calibration software. The resulting calibrated ^14^C dates are reported as age ranges. When viewed on the calendric time-scale, the corresponding multiple summing of the ^14^C-probabilities does not represent a dating probability in the traditional sense. This issue has been treated statistically in detail by Bronk Ramsey in the development of the OxCal program [72,90]. Using this program, we present for each investigated museum object a graphic figure of the calibrated radiocarbon age. The peaks have a higher probability, while the low parts of the graph have a lower probability. The median calibrated value is also reported as an additional characterization, signifying the central part of the total calibrated age range, i.e., 50% is older and 50% is younger. However, it has to be kept in mind that the median value of an irregular non-Gaussian calibrated age range does not necessarily represent a high probability. For bimodal distributions the median may even correspond to a time segment with low probability.

The comparative rarity of organic museum objects associated with the 17^th^ to early 18^th^ Dynasty transition period did not allow selection of samples that can be neatly arranged in a historical sequence. Therefore, Bayesian sequence analysis could not be used in our investigation. We took a different approach, which yielded meaningful results. Our ***uncalibrated*** radiocarbon dates of 17^th^/early 18^th^ Dynasty contexts are compared with a robust series of ***uncalibrated*** high-precision radiocarbon dates of the Minoan Thera eruption available in the literature.

It has to be kept in mind that ***uncalibrated*** radiocarbon dates in conventional ^14^C years BP constitute the primary measurement result of radiocarbon dating [106]. ***Calibrated*** radiocarbon dates are subject to ***change***, caused by ***revisions of the calibration curve*** [54,56,58–61,67,68,91,107]. However, ***the radiocarbon dates themselves in conventional*** ^***14***^***C years BP*** remain ***the same***. Relative dating within ^14^C time space can give meaningful results. Taking a significant group of individual radiocarbon dates of a certain event such as the Minoan Thera eruption, or of a historical segment of Egyptian history, such as the transition period from the 17^th^ to 18^th^ Dynasty, then each group of dates may show a distinct time signature in conventional ^14^C years BP. Such an approach facilitates judgement which group is older and which group is younger, even when the exact time in calendar years is not specifically addressed.

## Historical chronologies *before* and *after* Pharaoh Ahmose (II)

Radiocarbon dating by itself cannot determine the accession year and reign length of a king. Calibration into calendar years does not produce a precise point date, but a probability range. Therefore, historical chronologies form the basis of dynastic Egypt and its kings. Nevertheless, different interpretations of historical sources led to different chronologies. Here radiocarbon dating can make an important contribution, indicating which historical chronology over a certain period is to be preferred: “high, middle or low” [43,108]. Therefore, first an analysis is given of historical Egyptian chronologies relevant to the subject of our investigation, before presenting and discussing our radiocarbon dating results of museum objects derived from Abydos and Thebes (Fig 1), related to the 17^th^ Dynasty, Ahmose (II), and the early 18^th^ Dynasty.

Major literary sources about the history of ancient Egypt are Manetho’s Aegyptiaca [78] and the Papyrus Turin [109], but both have their limitations. The writings of Manetho are only known “*from fragmentary and often distorted quotations*” [78, p. viii]. The Papyrus Turin, written during the Ramesside Period, listing kings of Egypt with their length of reign, was discovered around 1823, but has since disintegrated into more than 300 fragments [109]. These fragments were rearranged as good as possible into columns and lines by Gardiner [110] and more recently by Ryholt [63], complicated by the problem of missing pieces and floating fragments. Other important sources comprise “hard” attestations of kings and high officials of the Second Intermediate Period found on monuments, stela, sculptures, tombs, seals, scarabs, and other archaeological objects [63,65,111–116]. Recent excavations in southern Egypt (Fig 1) at Abydos [111,112,116] and Tell Edfu [115] have uncovered particularly novel findings in this respect.

The 16^th^ and 17^th^ Dynasty were defined by Manetho, but transmitted confusingly by secondary sources. “*The Sixteenth Dynasty were kings of Thebes, 5 in number; they reigned for 190 years*” according to Syncellus, quoting Eusebius [78, p. 93]. However, quoting Africanus, a contradictory account is conveyed by Syncellus: “*The Sixteenth Dynasty were Shepherd Kings again, 32 in number; they reigned for 518 years*” [78, p. 93]. The term “Shepherd Kings” was already used by Africanus for the northern 15^th^ Dynasty [78, p. 91] and subsequently also for the 17^th^ Dynasty together with Theban kings: “*The Seventeenth Dynasty were Shepherd Kings again, 43 in number, and kings of Thebes or Diospolis, 43 in number. Total of the reigns of the Shepherd Kings and the Theban kings, 151 years*”, according to Syncellus [78, p. 95].

Winlock [117] suggested in 1947 to relate 6 kings known from epigraphic attestations to the 16^th^ Dynasty (Antef V, Rahotep, Antef VI, Antef VII, Sebekemsaf II, Djehuty) and 3 kings to the 17^th^ Dynasty (Senakhtenre, Seqenenre, Kamose). The latter 3 kings preceded the reign of Nebpehtire Ahmose, the first king of the 18^th^ Dynasty. However, Winlock [117] did not attempt to relate these 9 kings to the Turin King-list. This was done more recently.

Table 1 shows four scholarly associations of attested kings with the Turin King-list (TK) from column 10, line 30, until column 11, line 31 [63,118–120]. The structure of these TK columns in Table 1 is based on Allen [119], being virtually identical to the reconstruction by Ryholt [63], except for the last line, i.e., 11/35 in the latter and 11.31 in the former. Below this line the Papyrus Turin was cut away in ancient times and we can only speculate about its original continuation.

**Table 1:** The Turin King-list from column 10 line 30 to column 11 line 31 (end of papyrus) with associated interpretations of dynasties and kings. Reconstruction of the Turin King-list (TK) is based on Ryholt [63], slightly modified by Allen [119]. Tentative chronologies of kings (accession year or year of death BCE) are given by Franke [118] and Ryholt [63], who also suggested kings for blank TK lines. Wegner [120] mentioned preserved TK reign length years and a newly discovered king at Abydos, whose name Woseribre Senebkay [116,120] fits TK lines 11.16 or 11.17.

*Turin King-List* Allen [119]		Franke [118] 17^th^ Dynasty		Ryholt [63] 16^th^ Dynasty		Wegner [120] 16^th^ Dynasty	
16^th^ Dynasty							
[10.30] *[nyswt …]*							
[10.31]		Antef V	1625	unknown	1649	name lost	years lost
11.1	Djehuty or Rahotep	Rahotep	1622	Djehuty	1648	Djehuty	3 years
11.2	Sobekhotep VIII or Sobekemsaf I	Sebekemsaf I	1619	Sobekhotep VIII	1645	Sobekhotep VIII	6 or 16* years
11.3	Neferhotep III	Djehuty	1603	Neferhotep III	1629	Neferhotep III	1 year
11.4	Mentuhotep VI	Mentuhotep VII	1602	Mentuhotep VII	1628	Mentuhotep	1 year
11.5	Nebiriau I	Nebiriau I	1601	Nebiriau I	1627	Nebiriau I	26 years
11.6	Nebiriau II	Nebiriau II	1582	Nebiriau II	1601	Nebiriau II	
11.7	Semenenre	Semenenre	1582	Semenenre	1601	Semenenre	
11.8	Bebi-ankh	Bebi-ankh	(d) 1570	Bebi-ankh	1600	Bebi-ankh	12 years
11.9	Sekhemre-shedwaset	Sebekemsaf II	1570	‘Shedwast	1588	Sekhemre-shedwaset	
11.10		Antef VI		Dedumose I	1588	name lost	years lost
11.11		Antef VII	(d)1560	Dedumose II		name lost	years lost
11.12		Senakhtenre	1560	Montuemsaf		name lost	years lost
11.13		Seqenenre	(d) 1545	Mentuhotep VI		name lost	years lost
11.14		Kamose	1545	Senusert IV	(d) 1582	name lost	years lost
11.15 *nswt 5 jr.n*… *dynasty summation line*							
**17**^**th**^ **Dynasty**		**Unidentified Dynasty**		**Abydos Dynasty**		**Abydos Dynasty**	
11.16	Woser…re					Woseribre Senebkay	
11.17	Woser…					Woser…	
[11.18]						name lost	
[11.19]						name lost	
[11.20]						name lost	
[11.21]						name lost	
[11.22]						name lost	
[11.23]						name lost	
[11.24]						name lost	
[11.25]						name lost	
11.26	…heb					…hebre	years lost
11.27 *jr.n.f m nswyt*						name lost	2 years
11.28						name lost	2 years
11.29						name lost	4 years
11.30	…weben… I					…webenre	3-4 years
11.31	…weben… II					…webenre	3-4 years
**END OF PAPYRUS TURIN**							

Several scholars, including Franke [118], used the term “17^th^ Dynasty” to include all the known Theban kings between the late 13^th^ and 18^th^ Dynasty, as reviewed in detail by Schneider [121]. Franke [118] associated 15 Theban kings with Turin King-list 10/31–11/14. The next line TK 11/15 contains the phrase ***nswt 5 jr.n…***, being a summation line of the number of kings listed above (Table 1). The number 5 does not fit, but Von Beckerath [122,123] suggested that the original number must have been 15, which would indeed accommodate the number of lines for kings above the summation line, an interpretation supported by Ryholt [63]. The original TK text has not survived in TK 10/31 and 11/10–14, but Franke [118] also suggested kings for these 6 lines (Table 1). He estimated a cumulative reign length of about 86 years for the 15 Theban kings, from ca 1625 BCE until 1539 BCE, his preferred date for the accession year of Nebpehtire Ahmose and the beginning of the 18^th^ Dynasty. In addition, Franke [118] was the first to argue for a separate local Abydos Dynasty, but he did not attempt to relate such a dynasty to the Turin King-list.

Ryholt [63, p. 151–162] associated TK 10/31–11/14 with the Theban 16^th^ Dynasty (Table 1), also suggesting kings for lines without surviving names (TK 11/10–14). The 15 kings of the 16^th^ Dynasty reigned in his assessment for about 67 years from ca 1649–1582 BCE (Table 1). He assigned the remainder of the Turin King-list from TK 11/16–35 to the Abydos Dynasty [Ryholt [63], p. 163–166}, which he considered “*either contemporary with or later than the Sixteenth Dynasty*” [63, p.164]. Therefore, neither the 17^th^ nor the 18^th^ Dynasty can be found in the surviving part of the Turin King-list, according to Ryholt [63,109].

However, Allen [119] related TK 11.16 to 11.31 to the 17^th^ Dynasty (Table 1). These differences of opinion regarding dynastic association are also influenced by the lack of dynastic heading lines in this section of the Turin King-list. Therefore, it is not easy to make firm relations with Manetho’s 16^th^ and/or 17^th^ Dynasties. The above suggestion by Allen [119] seems less likely, because the 5 partially surviving names in this section of the Turin King-list cannot be associated with attested names of known 17^th^ Dynasty kings.

Archaeological excavations at South Abydos in 2014 uncovered the decorated tomb of king Woseribre Seneb-Kay, a hitherto unknown ruler of the Second Intermediate Period [116,120]. The name of this Abydos king fits the partial preserved text in the Turin King-list at 11/16 ***Woser…re*** and 11/17 ***Woser…***, as explained by Wegner [120, p. 298], see Table 1. The above discovery confirms the previously suggested association by Ryholt [63, p. 164–165] of these TK lines with the Abydos Dynasty, as well as the absence of the 17^th^ Dynasty in the Turin King-list.

Based on the Abbott Papyrus, dating to the reign of Ramesses IX, Winlock [64] made in the 1920s pioneering field investigations at Dra Abu el-Naga (Thebes) aiming to reconstruct the chronological order of 17^th^ Dynasty kings. Modern archaeological research here is continuing [124–128]. Concerning chronology, Ryholt [63] suggested tentative reign lengths for his sequence of 9 kings of the 17^th^ Dynasty (Table 2), but Polz [126, p. 218] stated: “*In the current state of knowledge, it seems impossible to assign even a vague number of regnal years to specific kings and hence to the entire dynasty – none of these rulers’ names can be identified on the last preserved page of the Turin King List*” (Table 2).

**Table 2:** Kings of the 17^th^ and early 18^th^ Dynasty and their estimated historical chronologies in years BCE. *Since the nomen of Senakhtenre is also Ahmose [65], we distinguish between the two Ahmose kings by adding (I) and (II) behind their names, as suggested by Cahail [66]. The mudbrick from the Temple of Ahmose (II), which we investigated, dates historically to ca year 22 of his reign [111,112].

17^th^ Dynasty Sequence of kings, Ryholt [63]			Chronology Ryholt [63]		Sequence of kings, Polz [125,126] Chronology impossible to determine	
*Nomen*	*Prenomen*				*Nomen*	
Rahotep	Sekhemre-wahkhau		1580–1576		Rahotep	
Sobekemsaf I	Sekhemre-shedtawy		1576–1573		Sobekemsaf II	
Antef VI	Sekhemre-wepmaat		1573–1571		Sobekemsaf I	
Antef VII	Nubkheperre		1571–1566		Antef VI	
Antef VIII	Sekhemre-heruhermaat		1568		Antef VII	
Sobekemsaf II	Sekhemre-wadjkhau		1566–1559		Antef VIII	
Ahmose (I)*	Senakhtenre		1559–1558		Ahmose (I)*	
Tao	Seqenenre		1558–1554		Tao	
Kamose	Wadjkheperre		1554–1549		Kamose	
Ahmose (II)*	Nebpehtire					
**Early 18**^**th**^ **Dynasty**	Breasted [88]	Wente & Van Siclen [129]	Shaw [75]	Schneider [130]	Hornung et al. [76]	Krauss & Warburton [89]
Ahmose (II)* Nebpehtire	1580–1557	1570–1546	1550–1525	1548–1523	1539–1515	1524−1499
*Accession Year*	*1580*	*1570*	*1550*	*1548*	*1539*	*1524*
*Conquest Avaris, ca year 18*	*ca 1562*	*ca 1552*	*ca 1532*	*ca 1530*	*ca 1521*	*ca 1506*
***Mudbrick, ca year 22***	***ca 1558***	***ca 1548***	***ca 1528***	***ca 1526***	***ca 1517***	***ca 1502***
Amenhotep I	1557−1501	1551–1524	1525–1504	1523–1502	1514–1494	1498–1477
Thutmose I		1524–1518	1504–1492	1502–1489	1493–1483	1476–1470
Thutmose II	1501−1447	1518–1504	1492–1479	1489–1476	1482–1480	1469–1468
Hatshepsut & Thutmose III		1503–1450	1479–1425	1476–1422	1479–1425	1468–1415
Amenhotep II	1448−1420	1453–1419	1427–1400	1422–1396	1425–1400	1415–1389

The number of attested Theban kings between the late 13^th^ and the end of the 17^th^ Dynasty increased from 9 in 1947 [117] to 15 in 1988 [118] and to 24 in 1997 [63]. More changes are likely in future research, as expressed succinctly by Marée [114, p. 241]: “*… many kings remain unplaced and without dynastic attribution, their names being attested in the epigraphic record but not preserved in the Turin King-list*”. A detailed investigation by Marée [114] of some 40 stela and statuettes led him to identify works made by the same artists at a sculpture workshop at Abydos. Thus, he concluded that the kings Rahotep Sekhemre-wahkhau, Wepwawetemsaf Sekhemre-neferkhau, and Pantjeny Sekhemre-khutawy probably ruled in that order shortly before the reign of Sobekemsaf II Sekhemre-wadjkhau. Concerning their dynastic attribution, Marée [114] considered these kings to belong either to the late 16^th^ or early 17^th^ Dynasty. However, Ryholt [63] and Wegner [120] suggested that Wepwawetemsaf and Pantjeny belong to the Abydos Dynasty.

The above uncertainties underline that historical chronological assessments of the 16^th^, 17^th^ and Abydos Dynasties in southern Egypt are rather tentative in the current state of knowledge. Different opinions exist, which will not be reviewed here, whether the 16^th^ Dynasty and the Abydos Dynasty were coeval from their beginnings or whether one preceded the other, and to what extent they developed during the late 13^th^ Dynasty or after the collapse of the 13^th^ Dynasty. Another question is the “boundary” between the 16^th^ and 17^th^ Dynasty, both in terms of timing and political cause? The bottom line is that the actual beginning of the 17^th^ Dynasty and its duration remain ambivalent, while the chronology of its attested kings, though tentatively suggested by Ryholt [63] cannot be determined according to Polz [125,126], due to lack of data (Table 2).

Concerning the sequence of the last three kings of the Theban 17^th^ Dynasty, there is general agreement: Senakhtenre Ahmose (I), Seqenenre Tao, and Wadjkheperre Kamose (Tables 1 and 2). The last king arising from this 17^th^ Dynasty family is Nebpehtire Ahmose (II). He reunited Upper and Lower Egypt following his victory over the northern 15^th^ Dynasty and the conquest of Avaris, approximately in year 18 [79,118] of his reign (Table 2). However, Manetho placed the beginning of the 18^th^ Dynasty at his accession year.

Estimations for year 1 of Ahmose (II) are usually based on historical data of successive reigns of kings (dead-reckoning) backward in time from the 26^th^ Dynasty, while considering possible overlapping coregencies, Sothic and Lunar data, as well as foreign synchronisms. Such assessments produced a variety of accession years for Ahmose (II), ranging from 1580 BCE [88] to 1524 BCE [89]. Six historical chronologies for kings of the early 18^th^ Dynasty are shown in Table 2 [75,76,88,89,129,130].

A unique chronology for the Second Intermediate Period was developed by Bennett [113], based on genealogical investigations of the governors of El-Kab, located ca 80 km south of Thebes (Fig 1). Successive generations of these governors can be synchronized with certain kings of the 13^th^, 16^th^ and 18^th^ dynasties [113]. Employing a minimal time-length reconstruction, Bennett [113] showed that at least 8 generations of El-Kab governors bridge the chronologically problematic part of the Second Intermediate Period.

The vizier Ay of El-Kab can be associated with the reign of the 13^th^ Dynasty king Merhetepre Ini (Table 3). This king is named in column 8, line 4 of the Turin King-list, being the 34^th^ king of the 13^th^ Dynasty [63, p. 73]. The continuous genealogy ends after 8 generations in the early 18^th^ Dynasty (Table 3): governor Renni of El-Kab died during the reign of Amenhotep I, the son of Nebpehtire Ahmose (II). Using a time frame of 25 years of government service by high officials per generation, based on Bierbrier [131], although this number may also be higher [132,133], the time length suggested by Bennett for the 8 generations of El-Kab governors is 8 x 25 = 200 years. Adopting again a minimal time-length approach, Bennett placed the death of governor Renni near the end of Amenhotep’s reign, which is ca 45 years after the accession of Nebpehtire Ahmose (II). This number has to be subtracted from the above 200 years, resulting in a ***minimum*** time distance of 155 years between year 1 of Merhetepre Ini to year 1 of Ahmose (Table 3).

**Table 3:** Minimum time length between the 12^th^ Dynasty king Senusert III (year 7) and the accession year of the first 18^th^ Dynasty king Nebpehtire Ahmose. Table based on Bennett [113, p. 240].

Time Distance between Kings	Minimum Time Length	Notes
From year 7 of Senusert III to the end of 12th dynasty	72 years	If reign of Senusert III is 19 years
Known 13th dynasty reigns to Merhetepre Ini	74 years	From Turin King-list
Other 13th dynasty kings	14 years	Assuming 1 year per king
From Merhetepre Ini to year 1 of Nebpehtire Ahmose	155 years	Estimate based on genealogies of the governors of El-Kab
From year 7 of Senusert III to year 1 of Nebpehtire Ahmose	315 years	

The Turin King-list has preserved the regnal time length of many 13^th^ Dynasty kings, for whom Bennett calculated a total of 74 years (Table 3). But 14 TK king lines of the 13^th^ Dynasty lack reign length data, particular after TK 8/8 [63], p. 73, 408], 4 lines after king Merhetepre Ini. Suggesting only one regnal year for each of these 14 kings (Table 3), Bennett [113] took again a minimalistic chronological approach. Going further backward in time to the Middle Kingdom, he calculated 72 years from the 7^th^ year of king Senusert III until the end of the 12^th^ Dynasty (Table 3), thereby adopting the now prevailing interpretation that Senusert III had a reign of 19 years [134]. The 7^th^ year of Senusert III is usually related to a heliacal rising of Sirius, as written on a papyrus from EI-Lahun (Berlin Museum Papyrus 10012) dated to the 19^th^ century BCE. Therefore, Senusert 7^th^ year is considered an astronomical chronological anchor in the Middle Kingdom. Various attempts have been made to calculate this Sothic date in relation to lunar observations recorded in other papyri of the 12^th^ Dynasty, as reviewed and reassessed by Rose [135].

In conclusion, Bennett’s historical genealogical chronometric studies provide a direct time link between the 12^th^ and the 18^th^ dynasty, independent of unresolved matters concerning the respective chronological relationships between the 13^th^, 15^th^, 16^th^ and 17^th^ Dynasties. Moreover, the genealogical time distance between year 1 of Merhetepre Ini to year 1 of Nebpehtire Ahmose is ***independent*** of the fall of Avaris and the archaeology of Tell el-Dab’a [18,136,137]. Bennett [113, p. 241] concluded that his ***minimalist*** chronometric studies (Table 3) support a ***high chronology*** for the Middle Kingdom and a ***low chronology*** for the beginning of the New Kingdom.

## Results and discussion

### The Ahmose mudbrick: Archaeological and historical context

**Fig 2:**
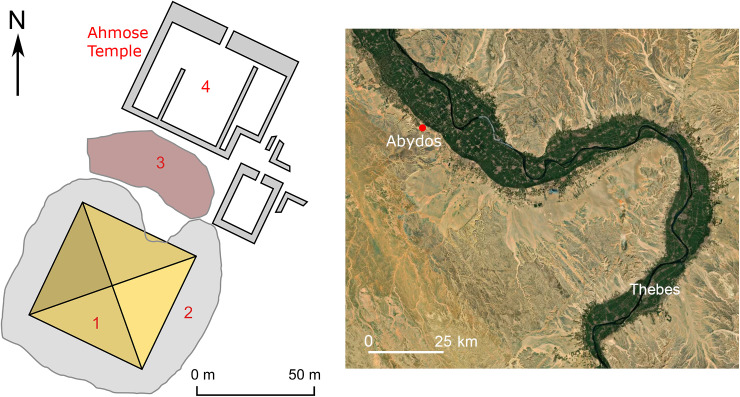
The Ahmose Temple and Pyramid complex at ancient Abydos in Upper Egypt west of the green Nile Valley. Plan of ancient buildings is from Wikimedia Commons open access: (1) Disintegrated Pyramid of Ahmose, (2) Hill of rubble, (3) Construction ramp, (4) The Ahmose Temple from where mudbrick EA 32689 is derived. Regional map of the Nile Valley (Abydos and Thebes area) is based on Mapcarta, the open map with CC BY license © OpenStreetMap, Mapbox, and Mapcarta.

The collection of the British Museum in London includes an unbaked clay brick bearing the stamped prenomen of Pharaoh Ahmose (II), i.e., Nebpehtire in hieroglyphic script. Since the name Ahmose was quite common during the late 17^th^ Dynasty, the prenomen, also termed cartouche name or throne name, makes the connection of the mudbrick with Pharao Ahmose (II) unmistakable. The mudbrick is derived from the excavations by Randall-MacIver and Mace [138] of the Ahmose Temple at Abydos (Figs 1 and 2) during their 1899–1901 campaign. The mudbrick was donated in 1900 to the British Museum by the Egypt Exploration Fund. Its registration number is 1900,1015.56 and the BM number is EA 32689.

Randall-MacIver and Mace reported that the mudbricks used in the construction of the Ahmose Temple “**were 16*½ *inches long, 7*½ *wide, and 5*½ *thick, and in most cases they were stamped with the name of the king (xxxii.)**” [138, p. 76, pl xxxii]. The dimensions of mudbrick EA 32689 in the British Museum, also derived from their excavations at the Ahmose Temple, are quite similar: 15½ inches long, 7½ wide, and 4½ thick. More recent excavations at the site were conducted by Harvey [111,112].

Mudbricks in ancient Egypt were produced in rectangular wooden frames (molds) without top or bottom. These empty frames were placed on a suitable flat landscape surface sprinkled with sand and straw to enable easy removal of the mudbricks after initial drying. The wet mud mixture was poured into the rectangular frames, which guaranteed the production of mudbricks more or less identical in size [96]. A number of possible causes may lead to variations in the size of mudbricks made in rectangular molds of equal size: a slightly uneven underground, non-uniform shrinkage upon drying, and some erosion during handling [139].

Yamamoto and Creasman [139] conducted an investigation about the size of mudbricks in relation to Dynastic history. The Middle Kingdom mortuary temple of the 12^th^ Dynasty king Senusert III at South Abydos was built with large mudbricks about 42 × 21 × 14 cm in size, while the associated town, also a royal initiative, used large bricks measuring about 39 × 19 × 12 cm [139]. The mudbricks from the Temple of Ahmose at Abydos have the following size ranges in centimeters, based on the above data by the excavators [138] and brick EA 32689 in the British Museum: 41.9–39.4 cm long, 19.1–19.0 cm wide, and 14.0–11.5 cm thick. These sizes are strikingly similar to the mudbricks used about 300 years earlier by Senusert III, also at Abydos. After defeating the Hyksos, Pharaoh Ahmose (II) may have been inspired by the architecture of the powerful Middle Kingdom at Abydos to build his own Temple, using mudbricks of similar size.

**Fig 3:**
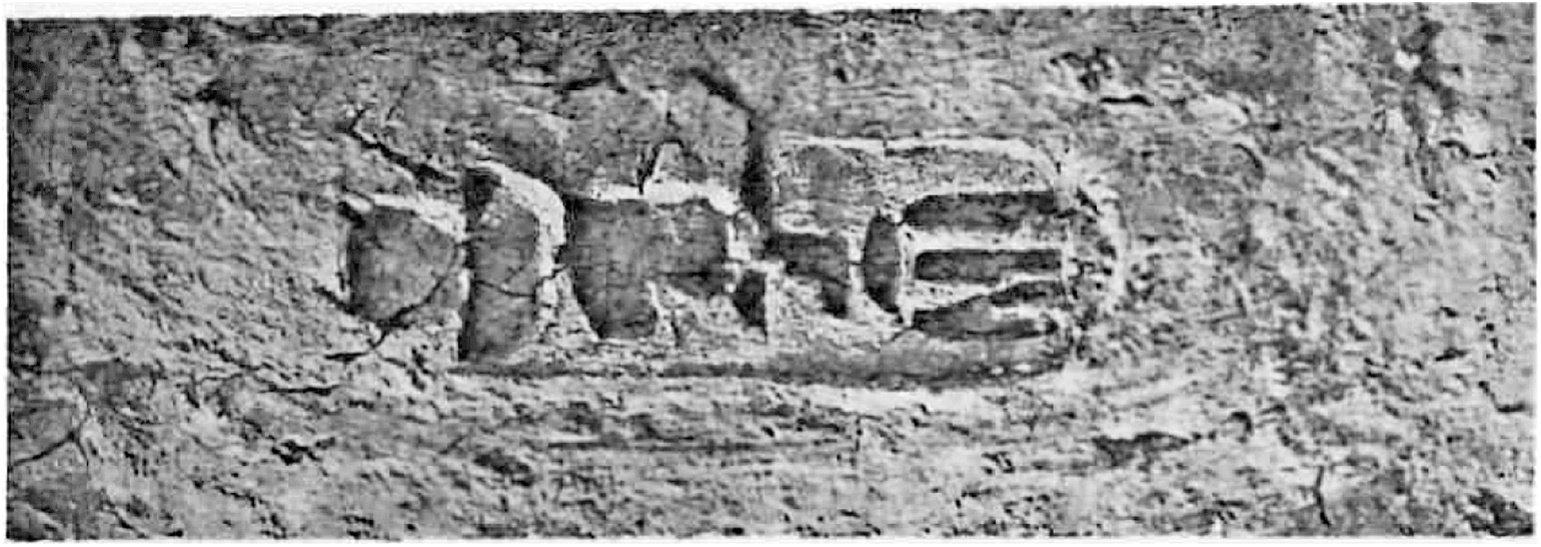
Brick with stamped prenomen Nebpehtire of Pharaoh Ahmose from the Temple of Ahmose at Abydos. Photograph from Randall-MacIver and Mace, 1902, Plate xxxii [138], reproduced under a CC BY license with permission and courtesy of © The Egypt Exploration Society, London.

**Fig 4:**
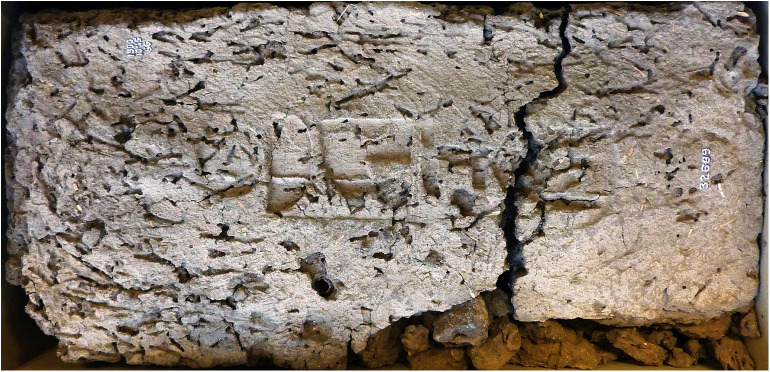
Mudbrick EA 32689 (British Museum) from the Temple of Ahmose at Abydos, showing the same stamped prenomen Nebpehtire of Pharaoh Ahmose. Photo by H.J. Bruins, 2018 © The Trustees of the British Museum, London. Shared under a Creative Commons CC BY-NC-SA 4.0 license.

Furthermore, within Egyptian Dynastic history the addition of a stamp on mudbricks began during the reign of Nebpehtire Ahmose [111,139,140]. Comparing the image (Fig 3) of a brick from the Temple of Ahmose published in 1902 [138] with the photograph of brick EA 32689 in the British Museum, taken by the first author (Fig 4), it can clearly be seen, notwithstanding the crack running through the latter mudbrick, that the stamped throne name Nebpehtire of Ahmose (II) is the same in both images.

The time link with Ahmose in our radiocarbon investigation is via mudbrick EA 32689, bearing his prenomen Nebpehtire. When was this brick made during his reign? Almost a century after the excavations at Abydos [138], a new archaeological survey of the Ahmose Pyramid complex (Fig 2) was initiated in 1993 by Stephen Harvey, who conducted various excavations that yielded important results [111,112]. The Ahmose Pyramid was as far as we know the last Royal Pyramid in Egypt, but the building disintegrated and only a mound of rubble survived (Fig 2). The Ahmose Temple was built adjacent to the Pyramid, on its north-eastern side (Fig 2). The excavations by Harvey [111,112] of the Ahmose Temple uncovered on its eastern side fragments of a battle narrative with horses and chariots, soldiers and ships. Hieroglyphic texts indicate these scenes to represent the battles of Ahmose against the Hyksos, as their capital Avaris (Fig 1) is mentioned in these texts. The new findings by Harvey clearly indicate that the construction of the Ahmose Temple and Pyramid occurred **after his victory over the Hyksos**, possibly during or after year 22 in his reign [111,112]. The year 22 of Ahmose is specifically recorded in the important Turah limestone quarries, which the king reopened [80,111]. Table 2 shows the following historical dating options for year 22 of Ahmose, when the mudbricks for his Temple were probably made: 1558 BCE [88], 1548 BCE [129], 1528 BCE [75], 1526 BCE [130], 1517 BCE [76]. 1502 BCE [89].

### The Ahmose mudbrick: Straw, color, and microscopy

**Fig 5:**
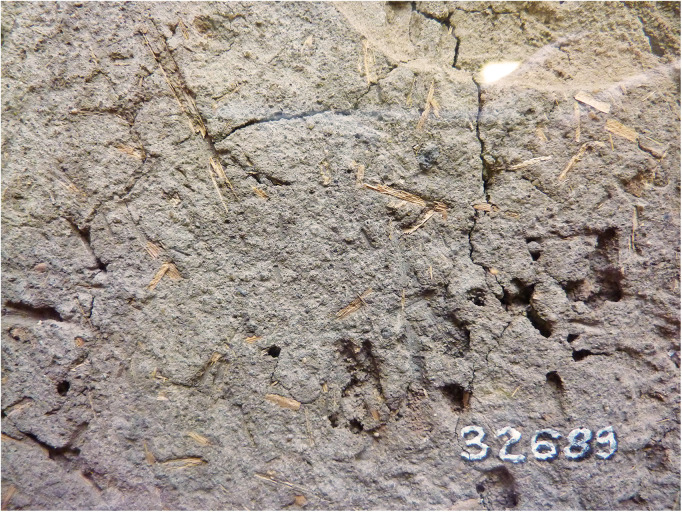
Plant fragments (straw) in the Ahmose mudbrick EA 32689 (British Museum). Photo by H.J. Bruins, 2012 © The Trustees of the British Museum, London. Shared under a Creative Commons CC BY-NC-SA 4.0 license.

Fragments of plant remains (straw) are clearly visible within the investigated Ahmose mudbrick (Fig 5). We also determined the color of the mudbrick, which is a significant characteristic. Its color can be categorized as greyish brown to dark greyish brown, 10YR 5/2–10YR 4/2, according to the Munsell soil color chart. Such a color fits type A mudbricks [96], usually made from fine-grained sediments deposited under low energy conditions and seasonal water logging, resulting in poor oxygenation.

Investigations to determine the species of plant remains (straw) in mudbricks are rare. We are not aware of any study on this subject concerning ancient Egyptian bricks. The only research in this field known to us is a study by Hendry and Kelly [141] about plant content of adobe bricks from buildings made by monks in Spanish California (1697–1821). The examined mudbricks were found to contain organic matter chopped to about 5 cm in length. “*Wheat and barley straw constituted the favorite organic material, but many other substances were employed, the choice apparently being determined by whatever was available at different seasons. Weeds of all kinds were extensively used, particularly those with fibrous stems, such as wild rye, sedges, tules, filaree, tarweeds, and various grasses, but the finding of other miscellaneous materials suggests that much of the general refuse from the mission was also utilized*” [141, p. 372]. These significant findings suggest that the term “straw” should not be limited to cereal grasses, but may refer also to other plants having fibrous stalks and stems.

**Fig 6:**
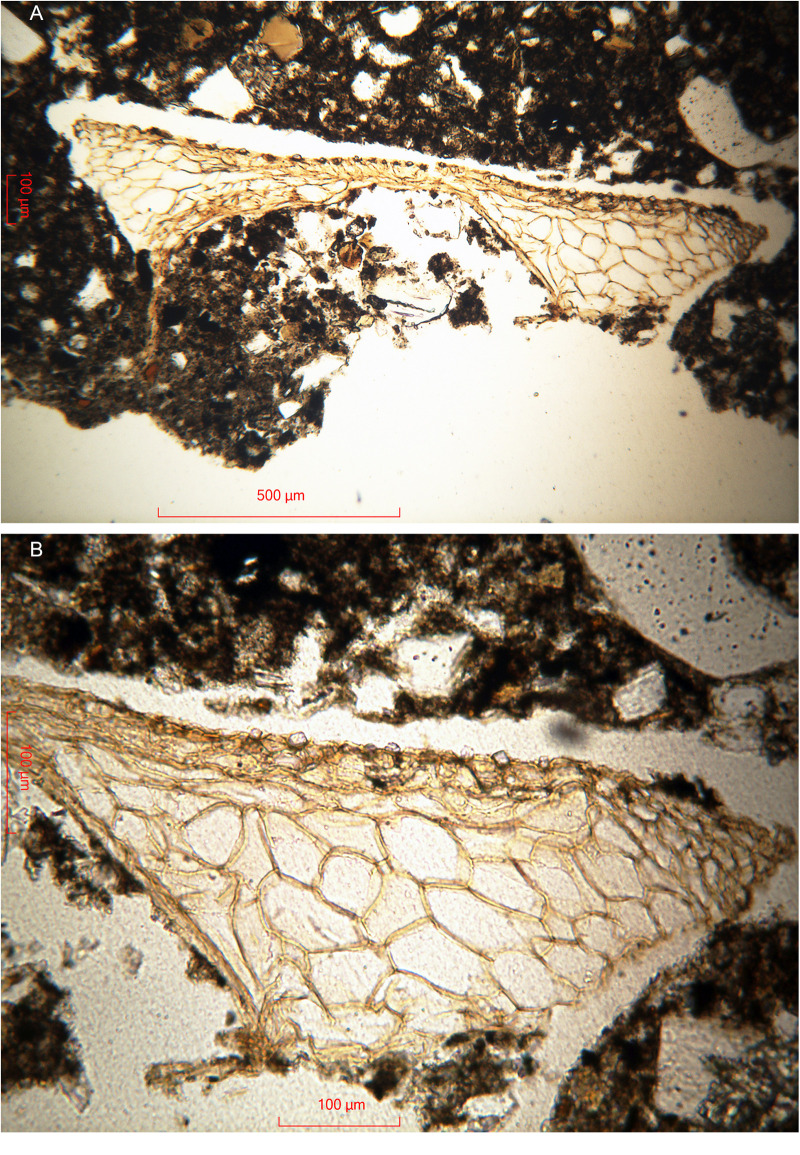
Thin section of an intact lump of the Ahmose mudbrick EA 32689. (A) Plant fragment, 1.4 mm long in plain polarized light. (B) Its right part in higher magnification to show the phytoliths within the epidermis. Microscope photos by H.J. Bruins, 2024 © The Trustees of the British Museum, London. Shared under a Creative Commons CC BY-NC-SA 4.0 license.

Which plants were possibly used in the area of Abydos (Fig 1) for adding straw in the production of mudbricks during the reign of Ahmose (II)? A thin section of the Ahmose mudbrick EA 32689 exhibited a number of plant remnants, often poorly preserved, due to desiccation and deterioration over time. Comparatively large voids within the mudbrick matrix may be the only memory of plant fragments that once occupied these spaces. However, one plant fragment in the mudbrick thin section displayed excellent preservation, facilitating botanical evaluation, though its length is only 1.4 mm (Fig 6).

Prof. Arlene M. Rosen (University of Texas at Austin, Department of Anthropology) kindly gave her expertise assessment regarding Fig 6. A large section of mesophyll tissue is visible, characterized by sizeable cells up to ca 100 micron, whereas the thin epidermis layer is situated at its upper part. The problem with identifying plant parts from thin sections is that the orientation is usually not ideal for an accurate identification. A top view of the epidermal tissue would have been better instead of the current side view. Nevertheless, small silicified cell bodies (phytoliths) are visible in the upper epidermis layer (Fig 6), which appear to be of a type often defined as “cones”, having a size of about 10 micron. If they are cones, the plant would be a sedge, i.e., belonging to the *Cyperaceae* family, with genera such as *Cyperus* and *Scirpus*.

Sedges have solid stems and narrow grasslike leaves, growing in marshy or irrigated grounds. They are used for matting, basketry, and straw [142–144]. The morphologically distinct conical shapes of phytoliths in sedge plants are present in the epidermal cells of leaves and stems [145]. Indeed, these parts of the plant, particularly the stems, could have been chopped up to provide straw for mudbrick fabrication. Conical phytoliths of leaves and stems may have a rounded, rectangular or square base [146]. The latter two shapes are actually visible with respect to the phytoliths in the epidermis of the microscopic plant fragment (Fig 6B) in the Ahmose mudbrick. A thin section gives of course a two-dimensional cut through the phytoliths, not showing their three-dimensional shape.

How did the 1.4 mm small sedge plant fragment end up in Ahmose brick EA 32689? There are two main possibilities. (1) It may have been derived from “fresh” living sedge plants chopped for straw at the time of mudbrick fabrication. The term “papyrus straw” [147] is not uncommon. (2) An “old” plant fragment already present in the seasonally wet soil, before its usage during the reign of Ahmose for making the mudbrick. In the latter case, the sedge plant fragment could be significantly older than the time of mudbrick fabrication, perhaps originating from sedimentary Nile debris or from human activities since Predynastic times. For example, all 1^st^ Dynasty kings and the last two kings of the 2^nd^ Dynasty were buried at Abydos, around 3000 BCE, in an area called Umm el-Qaab [148]. Mudbricks, obviously made from alluvial soils in the adjacent Nile Valley, were extensively used at this site in tombs, funerary enclosure walls, and temples.

### The Ahmose mudbrick: ^14^C and δ^13^C measurements

Radiocarbon dating of mudbricks, based on embedded straw fragments, added during the time of mudbrick fabrication, has given reliable results [97]. For example, straw in mudbricks and in mud mortar between limestone building stones of the Middle Kingdom 12^th^ Dynasty Pyramid of Senusert II at lllahun yielded radiocarbon dating results agreeable with the historical chronology [70]. However, more often the ^14^C dating results of organic material in mudbricks and mud seals were found to be older by many decades and even centuries than historical age assessments [70,71,97]. An explanation was suggested by Dee et al [97, p. 877]: “*It appears that the plant material already present in the mud itself was sometimes sampled for dating. Such fragments may be significantly older than their historical context, depending on their residence time in the original sediment*.” Based on the above findings and experience, the youngest ^14^C result within a series of radiocarbon dates from a specific mudbrick is more likely to represent the “fresh” vegetation added to the mud at the time of brick fabrication.

Concerning the Ahmose mudbrick, the sampling of clean straw without attached mudbrick material proved to be surprisingly difficult. The plant fragments are very brittle and strongly attached to the clayey mudbrick matrix. The surface of the mudbrick with the stamped prenomen Nebpehtire (Fig 4) shows plant fragments that resemble straw in terms of their yellowish color, shape and size: fibrous stems up to 0.5 cm wide and up to about 5 cm long (Fig 5). However, only one piece of pure straw, already partly loose, could be extricated successfully from the surface of the mudbrick, as destructive sampling is not allowed. This single pure straw fragment, sample GrA-64347, without attached mudbrick material, belongs to the largest plant size remains in the Ahmose mudbrick. The sample, although very thin, contained sufficient carbon to undergo full AAA pretreatment (Table 4). Its radiocarbon date of 3230 ± 60 BP (Table 4) is the youngest and most important result in the series of ^14^C measurements we obtained for the Ahmose mudbrick.

**Table 4:** Samples of Ahmose mudbrick EA 32689 with their δ^13^C values and uncalibrated radiocarbon results.

Sample #	Description	% C	Pretreatment	δ^13^C (‰)	^14^C date (BP)
GrA-64347	Pure straw	24.9	AAA	−12.4	3230 ± 60
GrA-59737	Straw & mud	46.9	AAA	−23.5	3290 ± 40
GrM-15973	Straw & mud	23.3	Acid only	−24.9	3285 ± 45
GrM-15201	Straw & mud	4.4	Acid only	−25.1	3385 ± 20
GrM-14176/14177	Straw & mud	Very small	Acid only	−25.6	3335 ± 75

The single piece of straw (GrA-64347) has a δ^13^C value of −12.4 ‰ (Table 4). Hence the straw is not derived from C3 cereal plants such as wheat or barley, but from a plant with C4 photosynthesis, which include the sedge family (*Cyperaceae*) and many (sub)tropical grasses. Moreover, a thin section of the Ahmose mudbrick (Fig 6) revealed the presence of a small plant fragment, 1.4 mm long, belonging to the *Cyperaceae* family. The sedges are the second most important C4 family, with approximately 1500 C4 plant species [149]. The *Cyperaceae* or sedges also constitute a major family in the Egyptian flora, composed of 47 species with many C4 plants [150], including papyrus (*Cyperus papyrus*). The hieroglyph symbol for sedge 𓇓 is also the symbol representing Upper Egypt. The sedge symbol occurs in one of the five titles of Pharaoh: “He of the Sedge and Bee” 𓆥, whereby the bee represents Lower Egypt. Both symbols combined define the Pharaoh involved as king of Upper and Lower Egypt [151]. Various δ^13^C values of ancient Egyptian papyrus, dated by the AMS labs at Oxford (OxA) and Vienna (VERA), range from −7.8 ‰ to −11.5 ‰ [152]. Fresh papyrus organic matter (*Cyperus papyrus*) from Lake Victoria in Kenya gave δ^13^C values of −13.45 ± 0.62‰ [153]. Our δ^13^C measurement, −12.4 ‰, of the pure straw sample (GrA-64347) from the Ahmose mudbrick sits in between these values.

The other samples consisted of mudbrick lumps, derived from an already disintegrated part of the Ahmose brick, visible in the lower right bottom part of Fig 4. These mudbrick lumps provided 5 samples for radiocarbon dating: GrA-59737, GrM-15973, GrM-15201, GrM-14176, GrM-14177 (Table 4). The δ^13^C values of these samples are typical for C3 plants. Besides cereals, there are also C3 sedge plants, which grow along the Nile riverbank, such as *Scirpus tuberosus Desf* having a δ^13^C value of −24.3‰ (OxA-16343) [154]. It is important to realize that the δ^13^C values of samples GrA-59737, GrM-15973, GrM-15201, GrM-14176, GrM-14177 resulted from a mixture of unknown plant fragments of various sizes (>0.2 mm), not from a single piece of straw. Therefore, their δ^13^C data do not represent a single plant species and could even be a mixture of a majority of C3 plants and a minority of C4 plant remains.

Visible plant fragments in these mudbrick lumps could not be extracted intact under dry conditions, as they disintegrated and pulverized into tiny pieces with mudbrick soil still remaining attached. All mudbrick lump samples were soaked in water and pretreated with hydrochloric acid (HCl). Only sample GrA-59737 was large enough, and not too delicate, to undergo full AAA pretreatment (HCl acid wash, followed by an alkali NaOH wash, and a final HCl acid wash). The various washings, also with pure water, were usually done over a sieve (filter) with openings of 0.2 mm (200 micron) in order to remove the very fine mud particles and concentrate the coarser particles including plant fragments of various sizes larger than 0.2 mm. Following pretreatment, sample GrA-59737 had a high carbon content of 46.9%. Its uncalibrated radiocarbon date is 3290 ± 40 BP, about 60 radiocarbon years older than the single pure straw sample GrA-64347 (Table 4).

The other 4 mudbrick lump samples (GrM-15973, GrM-15201, GrM-14176, GrM-14177) were too small or too fragile and received only the first pretreatment step (A). Sample GrM-15973 contained a significant amount of organic plant fragments of various sizes (>0.2 mm), resulting in a high carbon content of 23.3%. The uncalibrated radiocarbon date of GrM-15973 is 3285 ± 45 BP, virtually the same as the date 3290 ± 40 BP of the previous mudbrick lump sample GrA-59737 (Table 4).

Following pretreatment, sample GrM-15201 was found to have a low carbon content of merely 4.4% C. Therefore, the sample used for ^14^C dating must have contained a sizable amount of non-organic mudbrick soil particles, besides plant fragments and perhaps also soil organic carbon. Its uncalibrated ^14^C date, 3385 ± 20 BP, is about 100 radiocarbon years older than the two previous results from mudbrick lump samples, and about 160 years older than the single pure straw sample (Table 4).

Mudbrick samples GrM-14176 and GrM-14177 contained hardly any carbon. Their amounts of C could not be expressed in percentages and could not be converted into graphite for measurement by the Micadas AMS. Hence, their extremely low amounts of carbon were ^14^C dated as two aliquots of gas, whereby the ^14^C dating result, 3335 ± 75 BP, is in fact the combined average of both measurements. This result is about 100 radiocarbon years older than the ^14^C date of the pure straw fragment (Table 4).

Evaluating our 5 uncalibrated radiocarbon dates, obtained from different sub-samples of the Ahmose mudbrick, it is clear that the variance between the results is too large in order to consider their weighted average as representing the time of mudbrick fabrication. Indeed, the 5 dates combined do not pass the chi-square test. Hence, it seems that the Ahmose mudbrick contains organic carbon of different ages and origin, possibly including Nilotic debris and organic remains of previous human activities. The Abydos area has a long history of settlement since Predynastic times. The grayish color of the soil, used in the fabrication of the Ahmose mudbrick, indicate past hydromorphic environmental conditions (gley soils) that enhance the preservation of plant remains in the alluvial Nile soil, due to poor oxygenation.

The carbon content of alluvial loamy clay soils in the Nile Valley of central and southern Egypt is in the range of 1.5% to 2.7% [155]. Soil organic carbon is usually hundreds or even a few thousand years older than the live vegetation growing on the soil surface [156,157]. How can we differentiate between plant remains and soil organic carbon older than the time of mudbrick fabrication and “fresh” plant remains (straw) added in the process of making the mudbrick? It seems to be expected that the “fresh straw” will have a larger size than the older plant remains and soil organic carbon. Concerning the 5 dates we obtained of different sub-samples of the Ahmose mudbrick, 4 dates (GrA-59737, GrM-15973, GrM-15201, GrM-14176/14177) are derived from mud with mixed organic remains larger than the sieve openings of 0.2 mm. **Only one sample (GrA-64347) consisted of a single piece of straw without attached soil mud.** This straw fragment was a few cm long and up to 5 mm wide, belonging to the largest size of visible plant remains in the Ahmose mudbrick. Therefore, this largest single plant fragment, without attached soil mud, is regarded by us as representing the actual radiocarbon time of mudbrick fabrication(Table 4): 3230 ± 60 BP (GrA-64347).

The investigation by Bonani et al [70], which included radiocarbon dating of ancient Egyptian mudbricks, also produced concrete examples of large differences between the ^14^C date of a mudbrick lump sample and the separate ^14^C date of straw only, derived from the same mudbrick. The authors took mudbrick samples at Dashur from the Middle Kingdom Pyramid of king Amenemhet III (12^th^ Dynasty). Sample DRI-2948 consisted only of straw, collected from a mudbrick, giving a ^14^C date of 3442 ± 41 BP. On the other hand, a lump sample of the same mudbrick, DRI-2958, containing all organic constituents including straw, yielded a much older ^14^C date of 4452 ± 73 BP, a difference of about 1000 radiocarbon years! The authors added a footnote to sample DRI-2958: “*date includes older organic content in clay used for brick making*” [70], p. 1311). However, the ^14^C date of the straw (DRI-2948, 3442 ± 41 BP) is compatible with historical chronologies. We calibrated this result, using OxCal [72,90] with the latest calibration curve IntCal20 [59], yielding a 95.4% probability date of 1881–1626 cal BCE. Historical dates for the reign of king Amenemhet III range from a high of 1859–1813 BCE [158, p. xix] to a low of 1818–1773 BCE [79]. Hence, the calibrated radiocarbon date of the straw fits the above historical time options.

We include here a theoretical assessment to illustrate how “contamination” with older organic fragments may influence the radiocarbon date of mudbrick lump (bulk) samples in comparison with the ^14^C date of pure straw from the same mudbrick. A ^14^C date is obtained by measuring the so-called activity ratio ^14^a, which is the ratio of the ^14^C radioactivity of the sample and that of a reference material, oxalic acid [106]. The sample contains two groups of organic constituents: the “pure” material (straw added at the time of mudbrick fabrication) and “contamination” (older plant and organic fragments already present in the alluvial Nile soil used for mudbrick fabrication). In order to quantify the contribution of the “contaminant” to the ^14^C result of the “measured” mudbrick lump sample, we need to know the date of the “pure” straw material. In addition, we have to know the mass fraction of the “contaminant” and its ^14^C age. We use the following mathematical relation between the three ^14^a activities: (1) “measured” mudbrick lump sample, (2) “pure” straw, and (3) “contamination” consisting of other organic components:

^14^a(measured) = (1-f)^14^a(pure) + f ^14^a(contamination)

whereby f is the mass fraction of the contaminant, and (1-f) is the mass fraction of the pure straw. Thus, two unknown parameters have to be quantified: f and the ^14^a (or age) of the contaminating older organic material.

The “measured” Ahmose mudbrick lump sample in our case is GrA-59737. Its ^14^C date 3290 BP is “denormalized” using its δ^13^C value; 3290 BP is calculated using ^14^a_N_ = 0.6639 (i.e., normalized for fractionation to −25‰ according to the convention), which in turn is calculated from ^14^a = 0.6659, the measured activity ratio. We use for the “contaminant” in this example a ^14^C age of 5230 BP, which corresponds to an activity ratio ^14^a = 0.5215. Assuming 2% contamination, then f = 0.02. Now we have all required data for the above formula, which becomes:

0.6659 = (0.98) ^14^a (pure) + (0.02) (0.5215)

The resulting value for the “pure” straw sample is ^14^a(pure) = 0.6688, which results in a ^14^C age of 3231 BP, that is in fact GrA-64347, 3230 BP (Table 4).

Summarizing, the ^14^C date of the Ahmose mudbrick lump sample GrA-59737 is measured as 3290 BP. This result may be too old if the sample is contaminated with older material that could not be removed during pretreatment. Assuming a contamination of 2% older organic material with a ^14^C age of 5230 BP, the correct age of the mudbrick is 3230 BP; the “ageing effect” is 60 years. When we assume 5% contamination, this effect is about 100 years. These examples help to illustrate the ^14^C dating differences between the single piece of pure straw (GrA-64347) in comparison with the other mudbrick lump samples (GrA-59737, GrM-15973, GrM-15201, GrM-14176/14177).

### The Ahmose mudbrick: Calibration of the ^14^C measurements

**Fig 7:**
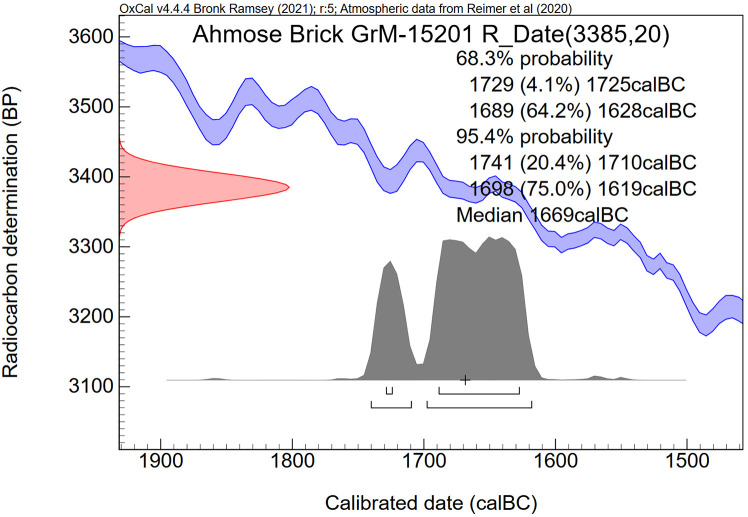
Calibrated ^14^C date of mudbrick lump sample GrM-15201 from the Ahmose brick.

Calibration of ^14^C dates into calendar years enables comparison of the Ahmose mudbrick with historical chronology options (Tables 2 and 3). Let us first calibrate the most precise ^14^C date we obtained, 3385 ± 20 BP (GrM-15201), derived from a mudbrick lump sample with a low carbon content of only 4% (Table 4). Using the OxCal program [72,90] with the IntCal20 calibration curve [59], the 68.3% probability calibrated age ranges (Fig 7) are 1729–1725 (4.1%), 1689–1628 (64.2%) cal BCE, while the broader 95.4% probability ranges are 1741–1710 (20.4%), 1698–1619 (75.0%) cal BCE.

These results of mudbrick lump sample GrM-15201 (Fig 7) are **much older** than all historical dating options (Table 2) for the reign of Nebpehtire Ahmose. The mudbrick from the Temple of Ahmose at Abydos (Fig 2) was most likely fabricated in year 22 of his reign [80,111,112], which would be 1558 BCE (Table 2) in the highest historical age assessment [88]. The calibrated radiocarbon date of GrM-15201 is older by 61–183 years (1741–1619 cal BCE). Therefore, mudbrick lump sample GrM-15201 is much older than the time of mudbrick fabrication, apparently due to “contamination” with older organic matter in the alluvial mud. The calibration result shows that a precise date is not necessarily an accurate date. Sample GrM-15201 can be safely rejected in relation to the fabrication time of the Ahmose mudbrick.

**Fig 8:**
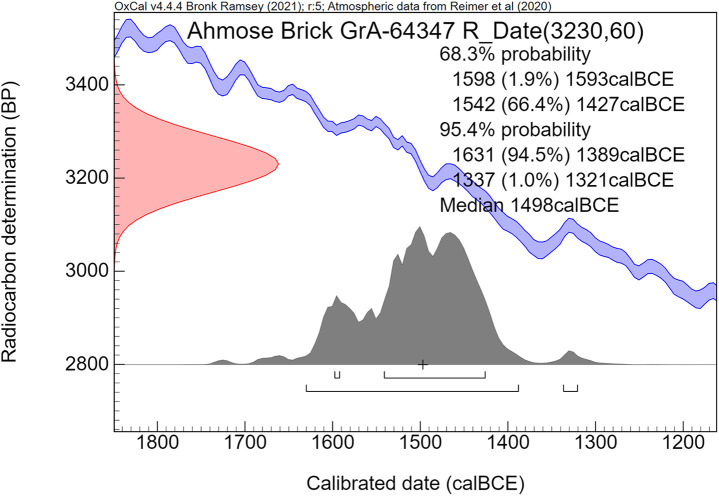
Calibrated radiocarbon age ranges of the pure straw fragment GrA-64347 from the Ahmose mudbrick.

Let us now consider the only radiocarbon date (GrA-64347, 3230 ± 60 BP) we have of a single piece of pure straw, which belongs to the largest plant fragments visible in brick EA 32689 (Fig 5). The comparatively large standard deviation of 60 yr BP results in a broad calibrated age range (Fig 8). Using OxCal [72,90] with IntCal20 [59], the age range 1542–1427 cal BCE (66.4%) has the highest probability, visually shown by the tallest peaks of the calibrated age graph (Fig 8). The center of these two peaks are positioned around 1500 cal BCE and 1470 cal BCE, respectively. Also the median value of 1498 cal BCE coincides with the highest peak.

Taking into account that mudbrick fabrication probably occurred around year 22 of Ahmose’s reign [80,111,112], the low historical chronologies by Krauss and Warburton [89] and Hornung et al. [76], respectively 1502 BCE [89] and 1517 BCE [76] for Ahmose year 22, are nearest to the calibrated radiocarbon date of both peaks. Such an indication is certainly significant. However, also other historical dating options for year 22 of Ahmose (Table 2) fit, with somewhat lower probability, the wide calibrated age range (Fig 8) of 1542–1427 cal BCE (66.4%), except for the two highest chronologies, i.e., 1558 BCE [88] and 1548 BCE [129].

Since sample GrA-64347 is a C4 plant, as shown by its δ^13^C value of −12.4 ‰ (Table 4), it may have been a sedge growing throughout the year in the riverine Nile valley in areas where there is enough soil moisture. Sedge plants and reeds are perennial, requiring soil moisture in every month of the year, so they will also grow in the late summer season when ^14^C in the air is at a maximum [159]. Therefore, we consider it inappropriate to make a minor correction for a possible reservoir effect that might have been caused if the plant would have grown only in the late winter season when ^14^C in the air is at a minimum [159]. Such a minor correction would have made our date even somewhat younger, because the IntCal20 and previous calibration curves are based on tree rings of wood that were growing in the northern hemisphere particularly during the summer ^14^C maximum [154,159,160].

Concerning the 4 mudbrick lump samples, their botanical plant content is unknown, except for a mudbrick lump that was used to make a thin section, whereby microscopic analysis showed the presence of a sedge plant fragment (Fig 6). There are also many other perennial reed-like grasses [161] in the Nile Valley that may have been used for providing straw in mudbrick production throughout the year. Therefore, we also consider it unjustified to make a minor correction for a possible reservoir effect regarding the mudbrick lump samples (GrA-59737, GrM-15973, GrM-15201, GrM-14176/14177), as we cannot know whether the unknown plant remains were only growing in the late winter season when ^14^C in the air is at a minimum [159].

**Fig 9:**
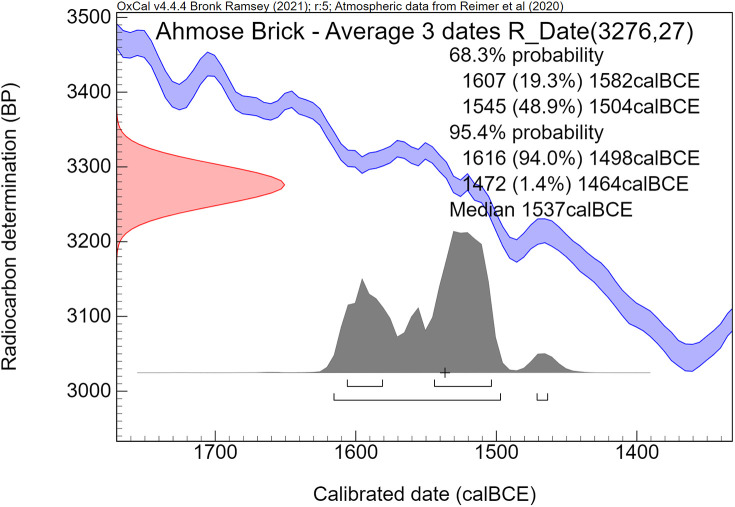
Calibrated age ranges of the weighted average of three radiocarbon dates of the Ahmose mudbrick, derived from samples with a high carbon content (Table 4).

Three radiocarbon dates are derived from samples with a high carbon content (Table 4): GrA-64347, GrA-59737, GrM-15973. Assuming that the two mudbrick lump samples (GrA-59737, GrM-15973) contained a significant amount of straw fragments, we may combine these dates with the ^14^C date of the pure straw sample (GrA-64347). The resulting weighted average date of 3276 ± 27 BP is statistically acceptable, passing the chi-square test: df = 2 T = 0.7(5% 6.0). Calibrating this average date results in the age ranges shown in Fig 9. The calibrated age with the highest relative probability, i.e., the highest peak, is 1545–1504 cal BCE (48.9%). The central part of the highest peak is situated around 1520 cal BCE (Fig 9). Year 22 of Ahmose in the historical chronology by Hornung et al. [76], i.e.,1517 BCE, is closest to this peak result, indicating again that our radiocarbon dating “straw” results of the mudbrick are most supportive of the younger historical chronologies regarding Ahmose. However, at somewhat lower probability, most historical Egyptian chronology options for year 22 of Ahmose (Table 2) fit this age range, except for the high chronologies [88,129]. The lower peak in the 1σ range (Fig 9) has a calibrated age range of 1607–1582 cal BCE with a probability of 19.3%. This time range, situated in the 17^th^ Dynasty, can be excluded as it is older than all historical age assessments for year 22 of Ahmose (Table 2).

Concerning the average of three radiocarbon dates of the Ahmose mudbrick (Fig 9), based on samples with a high carbon content, it must be emphasized again that the inclusion of two ^14^C dates containing a mixture of straw and mudbrick material probably gave a result that is too old, due to the likely presence in the mud of organic “contaminants” predating the time of mudbrick production. Therefore, we consider the radiocarbon age (Fig 6) of the single piece of pure straw (GrA-64347, 3230 ± 60 BP) to be the most reliable date for the Ahmose mudbrick, supporting a low chronology for the reign of Ahmose.

Radiocarbon dating corroborates the unique historical chronology investigation by Bennett [113], based on his genealogical study of the governors of El-Kab. Bennett was able to bridge a problematic part of the Second Intermediate Period, as detailed above (Table 3). He calculated a **minimum** time interval of 315 years between year 7 of Senusert III (12^th^ Dynasty) and year 1 of Nebpehtire Ahmose [113]. Such a block of time, which includes the Second Intermediate Period, can only be accommodated, according to Bennett [113, p 241], by “*a high chronology for the Middle Kingdom (year 7 of Senusert III= 1872 or 1866) and a low chronology for the New Kingdom (year 1 of Ahmose = 1539)*”.

Concerning Senusert III, there are a number of radiocarbon dating studies, supporting a high chronology for the Middle Kingdom [43,162,163]. The investigation by Bronk Ramsey et al. [43] included 10 high-quality radiocarbon dates in relation to Senusert III, which yielded the following modeled calibrated age ranges for his accession year: 1σ 1884–1860 cal BCE, 2σ 1889–1836 cal BCE. The historical dates suggested by Bennett [113] for year 7 of Senusert III are 1872 or 1866 BCE, both fitting very well within these radiocarbon dating results.

Our investigation of the Ahmose mudbrick EA 32689, stamped with his throne name Nebpehtire (Fig 4), provide the first ever radiocarbon measurements regarding his reign and the beginning of the 18^th^ Dynasty. Our calibrated radiocarbon date (Fig 8) of the large single piece of straw (GrA-64347), representing the time of fabrication of the Ahmose mudbrick from the Temple of Ahmose at Abydos (ca year 22 of his reign), supports a low chronology for year 1 of Nebpehtire Ahmose.

## Linen burial cloth associated with Queen Satdjehuty

### Archaeological and historical context

Satdjehuty was the daughter of Pharaoh Senakhtenre Ahmose [65] and Queen Tetisheri, who were the grandparents of Pharaoh Nebpehtire Ahmose (Table 2). Satdjehuty became queen as the second spouse of the next 17^th^ Dynasty king Seqenenre Tao in the region of Thebes in upper Egypt. He seemed to have opened the war against the Hyksos 15^th^ Dynasty and eventually died in battle, as indicated by the severe wounds visible in his mummified head. The senior spouse of Seqenenre Tao was Queen Akhotep I. They were the parents of Nebpehtire Ahmose [63].

The burial remains of Satdjehuty were discovered around 1820, including the famous gilded cartonnage mummy mask (EA 29770) and various linen burial cloths (mummy-wrappings), kept in the British Museum in London. Unfortunately, both the mummy and coffin apparently got lost, but the coffin lid, made of gold-plated sycamore wood and stucco, is now held in the State Museum of Egyptian Art in Munich.

The actual finding spot of Satdjehuty’s burial remains unknown, as no data exist about their discovery around 1820. Perhaps the necropolis of Dra Abu el-Naga, located west of the Nile at Thebes (Fig 1), may be a possibility [124]. Evaluating the association of Satdjehuty with burial cloth EA 37106, it has to be kept in mind that there is a gap of about 60 years between the discovery of Satdjehuty’s burial remains ca 1820 and the purchase of the mummy mask and linen mummy-wrappings by the British Museum in 1880 from Morten & Son. The items were inspected by Samuel Birch, who suggested that the mask and the textiles had belonged to the same person, i.e., Satdjehuty (Minutes of the British Museum Trustees Standing Committee, 8 May 1880). The acquisition notes by the British Museum inform that the items were “*From the sale of the collection of Samuel Hull of Uxbridge (c. 1799-1880). The mask, together with other objects, had probably been obtained by Samuel Hull’s brother, John Fowler Hull (1801-1825) during his visit to Egypt in 1824 (as noted by his fellow-traveller John Madox)*”.

The splendid mummy mask with a golden skin shows that Satdjehuty was a woman of the highest rank in the royal family [164,165]. Some of the linen mummy-wrappings bear inscriptions, even mentioning the name of Satdjehuty, while others do not. For example, a hieroglyphic inscription in red pigment appears on a fragment of a linen mummy-wrapping, stating “*Given in the favour of the god’s wife, king’s wife and king’s mother Ahmose Nefertari may she live, so Satdjehuty*” [166]. The text seems to imply that the linen cloth was donated for the burial of Satdjehuty by her niece Queen Ahmose-Nefertari, the wife of Nebpehtire Ahmose. Concerning Satdjehuty’s mummy mask, Strudwick [164] noted: “*The feather effect of the winged headdress on this mask should perhaps be associated with the so-called rishi-type coffin popular in Thebes at the very end of the Second Intermediate Period and the early New Kingdom*.” The name ‘rishi’ is derived from the Arabic word for feather, ‘risha’. This type of funerary coffins was investigated in detail by Miniaci [167].

When did Queen Satdjehuty die? She was born during the 17^th^ Dynasty, one generation before Nebpehtire Ahmose [63]. Concerning the time of her death, the texts written on one of her linen mummy-wrappings mention Queen Ahmose-Nefertary, the spouse of Pharaoh Nebpehtire Ahmose, as the “king’s mother”. Therefore, it seems that Amenhotep I, the son of Ahmose and Ahmose-Nefertary, had already become king before Satdjehuty died. Historical dating options for the accession year of Pharaoh Amenhotep I (Table 2) range from a high of 1557 BCE [88] to a low of 1498 BCE [89].

### Radiocarbon date of Satdjehuty’s linen burial cloth EA 37106

The British Museum agreed to provide a small fragment of a linen mummy wrapping (EA 37106; Registration number 1880,0521.12), associated with Queen Satdjehuty, for radiocarbon dating. This linen burial cloth is characterized by a plain warp fringe, but does not have an inscription. Its dimensions as a bundle are 23 cm long and 16 cm wide. The sample was taken by staff of the British Museum on 24 September 2013 in the presence of the first author.

The linen textile fragment contained a large amount of carbon (42.4%), underlining the quality of the material (Table 5). The sample was measured twice (duplo) in order to increase dating reliability and to obtain a weighted average result with a small standard deviation: 3310 ± 25 BP (GrA-59770).

**Table 5:** Radiocarbon dating of linen burial cloth EA 37106, associated with Queen Satdjehuty.

British Museum Nr.	% C	Pre-treat-ment	δ^13^C (‰)	Groningen Sample Nr.	^14^C Date (yr BP)	68.3% Calibrated Age (cal BCE)	95.4% Calibrated Age (cal BCE)	Median Value (cal BCE)
EA 37106	42.4	AAA	−25.0	GrA-59770	3310 ± 25	1612–1573 (39.8%) 1566–1538 (28.5%) *Including seasonal offset 12 ± 5 BP* 1611−1574 (36.1%), 1563−1553 (10.1%), 1548−1531 (17.1%) 1525−1519 (5.0%)	1624–1510 (95.4%) *Including seasonal offset 12 ± 5 BP* 1617−1508 (95.4%)	1574

**Fig 10:**
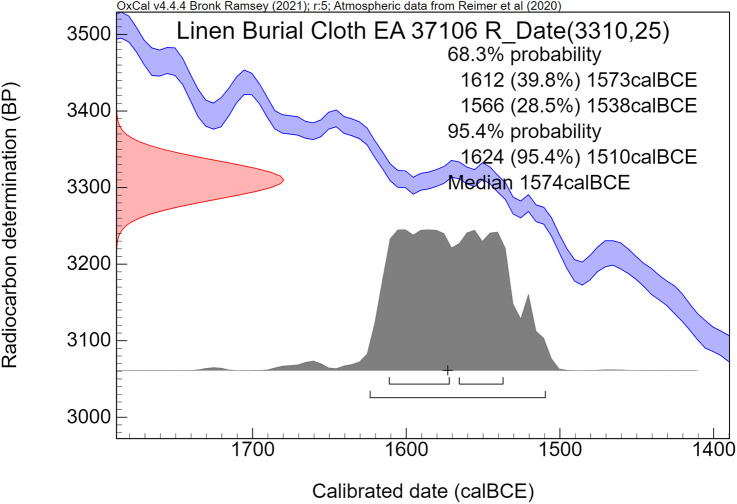
Calibrated radiocarbon age ranges of linen burial cloth EA 37106, associated with Queen Satdjehuty.

Linen is derived from fibers inside the stalks of the flax plant *Linum usitatissimum*. Flax is an annual plant sown in ancient Egypt during autumn and harvested in March or April. Therefore, part of its growth occurs during the late winter season when the ^14^C content in the air shows minimum values [159]. *Linum usitatissimum* was not included in the pioneering investigation by Dee et al [154] about a possible seasonal radiocarbon offset for ancient Egypt. Concerning the plants studied the offset showed large variations in radiocarbon years from +56 years BP to –40 years BP [154, p. 689]. The recommended “average” figure for the offset has been recalculated by Manning et al [160] for IntCal20 as ca 12 ± 5 ^14^C years. Besides the regular radiocarbon calibration (Table 5, Fig 10) of our ^14^C date for the linen burial cloth EA 37106, we also show the calibrated age ranges calculated with inclusion of the above seasonal offset of 12 ± 5 ^14^C years. The difference between the two calibration results is very small indeed; the seasonal offset lowers the calibrated date by only a few years (Table 5).

The standard calibrated age graph (Fig 10) shows one broad peak with two age ranges within 68.3% probability: 1612–1573 cal BCE and 1566–1538 cal BCE. Both age ranges have a significant relative probability, 39.8% and 28.5%, respectively. The oldest calibrated age is coeval with the 17^th^ Dynasty and the younger age range relates either to the 17^th^ or 18^th^ Dynasty, depending on the accession year of Nebpehtire Ahmose. Our radiocarbon dating results of the Ahmose mudbrick, coupled with Bennet’s [113] historical chronology, would also place the younger calibrated age range of burial cloth EA 37106 in the 17^th^ Dynasty.

Given the historical indication that Satdjehuty probably died during the reign of Amenhotep I, it is striking that the 68.3% probability radiocarbon dating result of Satdjehuty’s burial cloth is significantly older than most historical dating options for the reign of Amenhotep I (Table 2). In fact, the uncalibrated date is about 80 radiocarbon years older than the ^14^C date of straw (GrA-64347, 3230 ± 60 BP) in the mudbrick of Ahmose, the father of Amenhotep I. Therefore, our radiocarbon dating results of burial cloth EA 37106 raise a number of questions: (a) Perhaps the linen cloth was a special 17^th^ Dynasty heirloom given for the burial of Satdjehuty in the early 18^th^ Dynasty? (b) Perhaps Satdjehuty died earlier than generally inferred from the above mentioned inscription on another mummy wrapping? (c) Perhaps the association of burial cloth EA 37106 with Satdjehuty is incorrect? Notwithstanding the above questions concerning mummy wrapping EA 37106, its calibrated 2σ dating range, 1624–1510 cal BCE (95.4%), unquestionably covers the 17^th^ Dynasty and its transition to the beginning of the 18^th^ Dynasty.

## Petrie’s 17^th^ Dynasty wooden stick shabtis from Thebes

### Archaeological and historical context

The Petrie Museum of Egyptian and Sudanese Archaeology, University College London, has a collection of 44 wooden stick shabtis, which have been described and photographed in detail by Whelan [95]. Sir Flinders Petrie acquired these shabtis at Thebes in the late 19^th^ and/or early 20^th^ century. Petrie stated in his book about Shabtis [94, p. 3]: “*a large number of figures have been found at Thebes, which are evidently from a family cemetery, shortly before the 18*^*th*^ *dynasty. The only description at first hand is by Newberry in Excavations in the Theban Necropolis. Scattered ones have come through dealers to the British Museum, and at Thebes I bought about forty… These are all of wood, roughly split and chopped, and some even show no difference between head and feet. Yet they retain the old formula, and represent mummies*”.

**Fig 11:**
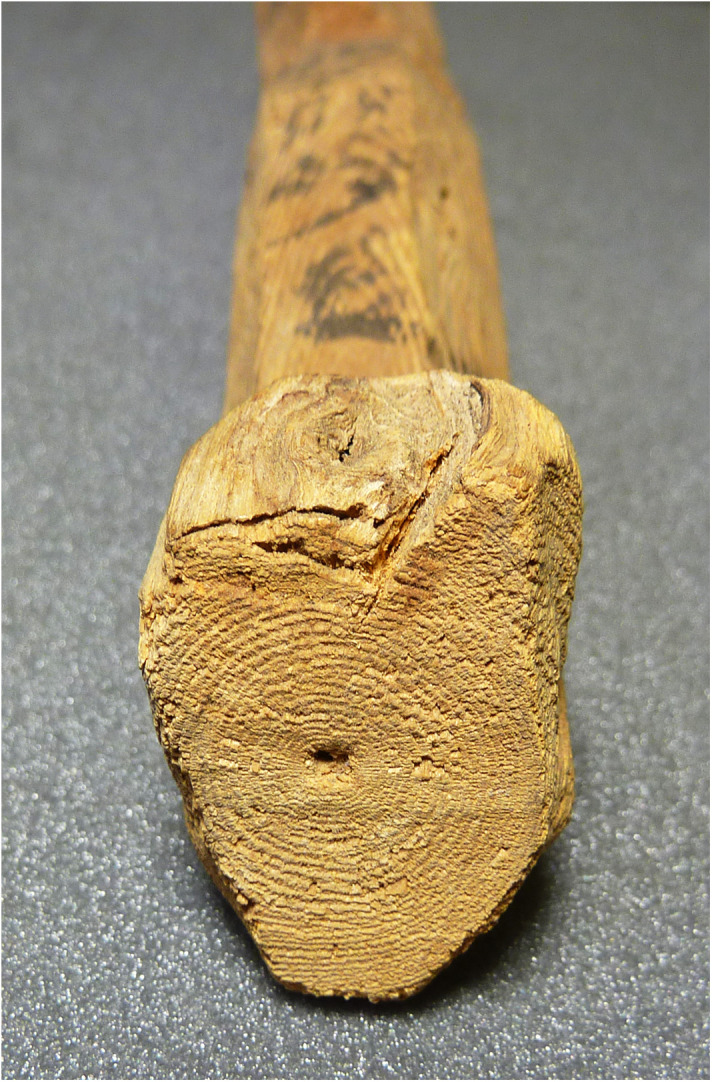
Tree rings are clearly visible in the bottom part of Shabti UC 40184 (diameter 3.1 cm). Photo by H.J. Bruins (2017), published with permission from the Petrie Museum of Egyptian and Sudanese Archaeology (University College London) under a CC BY license.

The Petrie Museum kindly allowed inspection of these shabtis, in order to evaluate their suitability for radiocarbon dating. The type of wood (Fig 11) has so far not been investigated botanically, but judged by their appearance, Whelan [95] considered that most shabtis in the collection of the Petrie Museum were made of the same species. All 6 shabtis investigated by us were made of soft wood, light yellow-brown in color. (Fig 11). The most likely candidate is wood from *Ficus sycomorus*, which had a widespread distribution in Egypt. The sycamore fig tree is mentioned in many ancient Egyptian texts and was considered one of the most important fruit trees [168]. An investigation of wooden Egyptian coffins in the British Museum revealed that *Ficus sycomorus* wood was used in the making of all seven coffins from Thebes belonging to the Second Intermediate Period [169].

Staff members of the Petrie Museum took tiny wood samples of a number of shabtis for radiocarbon dating, in the presence of the first author, on 10 September 2017. The paramount consideration of the museum staff was of course to select wood splinters from the bottom part of the shabtis that would not damage the ancient object. Nevertheless, care was taken to collect wood splinters from the outer tree rings, if achievable, in order to obtain radiocarbon dates as close as possible to the actual time of shabti production.

It should also be realized that the shabtis have a rather small width or diameter of only a few centimeters, as shown in Fig 11, which has a diameter of 3.1 cm [95]. Hence, the ancient shabti producers usually selected small tree branches, having the required size for making these type of stick shabtis. Small branches do not have many tree rings. Thus, the so-called “old wood effect” is probably minor, in the range of 1–15 years, as outer tree rings could generally be selected in the sampling procedure for radiocarbon dating. Such an “old wood effect” is smaller than the uncertainty (standard deviation σ) of the ^14^C measurement. The *Ficus sycomorus* is a large tree, having many branches that spread out more or less horizontally to a radius of 15–20 m, already beginning from the lower part of the trunk [170]. Indeed, also from this perspective, the *Ficus sycomorus* tree can provide many sticks for making shabtis.

The six radiocarbon dated shabtis have Petrie’s classification numbers 11, 12, 14, 15, 17, 29, respectively. These shabtis, attributed by him to the 17^th^ Dynasty [94], are presented and discussed below with their respective ^14^C and δ^13^C measurements. All these shabtis have the royal offering formula or plain names written in rude hieroglyphics on their front, side parts, or back [94,95]. The first five investigated shabtis may belong, according to Petrie [94], to a group of one family of six brothers, sons of Antef and Sat-art. However, this is not certain, because the archaeological context of each shabti is unknown, except that they came from the Thebes region. The name Antef was quite common in the 17^th^ Dynasty, as three of its pharaohs (Table 2) bear this name [63,125].

### Radiocarbon dating and δ^13^C values of six wooden stick shabtis

The dated fraction of all six shabtis is holocellulose. The amount of carbon (C) in each sample was found to be high (Table 6). The stable carbon isotope ^13^C measurements, which are necessary to account for fractionation in radiocarbon dating, also give important information regarding the possible type of tree species used for making the shabtis. The δ^13^C values of the six shabtis range from −26.6 ‰ to −29.3 ‰ (Table 6). These values seem to match quite well with δ^13^C data of ancient *Ficus sycomorus* samples from Egypt, −27.6 ‰ and −25.7 ‰, measured at the Oxford laboratory [171]. Alternative tree species in ancient Egypt used for shabtis could have been *Tamarix* or *Acacia* [95]. However, both of these trees have δ^13^C values that are less negative. For example, the few *Tamarix* trees growing today at Mount Sedom near the Dead Sea in Israel, a hyper-arid region with 46 mm average annual rainfall (1960–2005), have δ^13^C values ranging from −21.1 ‰ to −22.7 ‰. Tree rings of ancient *Tamarix* wood uncovered at Mount Sedom have δ^13^C values in the range of −25.8 ‰ to −20.6 ‰, as wetter periods in the past were found to be related to more negative δ^13^C values [172]. Measurements of ancient *Acacia* wood from Egypt yielded similar δ^13^C results in the range of −24.1 ‰ to −21.5 ‰ [171]. Therefore, it may be concluded on the basis of their δ^13^C values that the six investigated shabtis are not composed of *Tamarix* or *Acacia* wood, but were made from *Ficus sycomorus* wood.

**Table 6:** Radiocarbon dating results of six wooden stick shabtis from the Petrie Museum.

Wooden Stick Shabti: Petrie#, UC#, Name	Pre-treat-ment	% C	δ^13^C ‰	Groningen Lab Nr.	^14^C Date (yr BP)	Calibrated Age Range 68.3% (cal BCE)	Calibrated Age Range 95.4% (cal BCE)	Median Value (cal BCE)
#11, UC 40178 Teti-sa-intef	ABA	37.2	−26.9	GrM-31118	3295 ± 28	1610–1575 (32.3%) 1563–1518 (35.9%)	1620–1505 (95.4%)	1563
#12, UC 40179 Tet-sa	ABA	46.9	−26.2	GrM-12680	3224 ± 30	1510–1447 (68.3%)	1535–1426 (95.4%)	1479
#14, UC 40181 Teti-sa-intef	ABA	49.2	−29.3	GrM-12683	3185 ± 30	1497–1474 (29.8%) 1497–1430 (38.5%)	1506–1411 (95.4%)	1459
#15, UC 40182 Teti-sa-intef	ABA	49.6	−26.8	GrM-12684	3288 ± 30	1609–1577 (28.1%) 1561–1554 (5.5%) 1546–1510 (34.7%)	1622–1500 (95.4%)	1557
#17, UC 40184 Teti-sa-intef	ABA	48.5	−26.6	GrM-12685	3251 ± 30	1537–1495 (52.0%) 1477–1456 (16.3%)	1611–1574 (10.8%) 1565–1442 (84.6%)	1512
#29, UC 40196 Djehuty	ABA	38.8	−28.5	GrM-31119	3300 ± 30	1612–1573 (37.1%) 1566–1532 (31.2%)	1629–1502 (95.4%)	1567

The *Ficus sycomorus* tree, indigenous in Africa, grows near rivers, streams, drainage lines, springs and in areas where the water table is high. It is a large tree with many branches, forming an umbrellalike shape that provides welcome shade in a hot climate [173,174]. Concerning the calibration of our radiocarbon dates of the shabtis, we did not apply a minor correction for a possible reservoir effect [154,159,160], because the *Ficus sycomorus* tree grows only in places where there is enough water throughout the year [173, 175]. Therefore, its growth in ancient Egypt was not limited to the late winter season when ^14^C in the air is at a minimum [159]. In fact, along the Mediterranean coast of Israel, the sycamore tree may shed most of its leaves during cold winters [175], indicating that its growth is rather limited during the winter season. Although there is no rain in Israel during the long summer season, from about May to October, ground water is within reach of the tree roots. Indeed, the sycamore trees in Israel produce ca 3–7 generations of fruits (syconia) during the summer and early autumn, but during the winter fruits grow very slowly and remain green and hard for a long time [175].

In ancient Egypt, the sycamore was regarded as the tree of life, mentioned in many religious and historical texts [168]. Its remains are already found in predynastic times, but appear more abundantly in tombs of the Old Kingdom and subsequent dynastic periods, including sycamore fruit, timber, and even twigs [176]. The sycamore tree, providing fruits and shade, was very popular in ancient Egyptian gardens, planted around artificial pools, as the tree requires water during all months of the year. An example of such a garden in ancient Thebes, is recorded on the 11^th^ Dynasty stela of Samentuser (Florence Museum). The translation of the text on his stela reads: “*I am one with beautiful pools and tall sycamores*” [168, 177, p. 25].

The area of Thebes, from where the shabtis we investigated originate, is characterized by high groundwater levels in the Nile Valley [178,179], facilitating irrigation throughout the year, even in ancient times, to sustain horticulture and the presence of *Ficus sycomorus* trees. A report from the Thebes area in the 1940s concerning the peasant village of Gourna, situated between the ancient cemetery of Dra Abu el Naga and the Nile, states: “*subsoil water rises every year to within two meters of the surface*” [179, p. 179]. This was a common feature for thousands of years before the completion of the Aswan High Dam in 1968. Indeed, the ancient wealth of the Thebes region may be related to the presence of groundwater resources close to the surface, enabling irrigation of crops and trees at any time of the year [180].

The radiocarbon dating results (Table 6) are evaluated and discussed in detail for each individual shabti. In addition, the English translation of ancient Egyptian texts on the respective shabtis are quoted from Whelan [95], as well as other relevant information. The transliteration of ancient Egyptian names into Latin script is not uniform, related also to different pronunciations in English, French, and German. Concerning the shabtis, we generally use the spelling by Petrie [1935], but quotations from Whelan [95] show slight variations in comparison with the former. For kings we generally use the transliteration as given in Tables 1 and 2.

### Wooden stick shabti UC 40178

**Fig 12:**
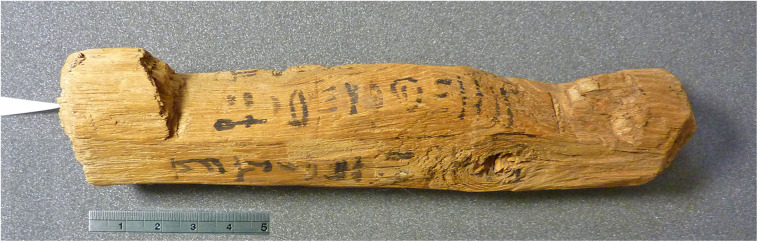
Shabti UC 40178. Photo by H.J. Bruins (2017), published with permission from the Petrie Museum of Egyptian and Sudanese Archaeology (University College London) under a CC BY license.

Petrie attributed Shabti UC 40178 to the 17^th^ Dynasty. This shabti is nr. 11 in his classification [94]. Its dimensions are 16.4 cm high, 3.2 cm wide, and 3.6 cm in diameter, as published by Whelan [95]. The white arrow indicates that tiny wood splinters for radiocarbon dating were taken from the bottom (feet) part of the shabti (Fig 12). Its head type “*roughly carved with a large nose and deep-set eyes*” [95, p. 71], cannot easily be assigned to one of the 11 defined shabti head styles [95]. Four columns of black hieroglyphs, separated by single black lines, appear on the front, back and both sides of this crude mummiform shabti [95].

Petrie catalogued each respective shabti with a number and with the name of the deceased person, which in this case has been transliterated by him as Teta-sa-antef [94]. The same name, or a variety thereof, occurs on Petrie’s shabti numbers 10–17, of which nr. 11, 12, 14, 15, and 17 are included in our radiocarbon dating investigation. Teta-sa-antef also occurs on other shabtis kept in museums in Leiden (# 2.1.1.4) [181] and Cairo (# 47909 and 47911) [95].

Shabti UC 40178 was dedicated to the deceased person Teta-sa-antef by his mother Sat-art. The full hieroglyphic text on this shabti has been translated by Whelan [95, p. 72]: “*An offering which the king gives to Ptah (and) Sokar so that they may give everything good and pure which a god lives on for the ka of Teti-sa-intef (by) his mother Sat-irt*”.

**Fig 13:**
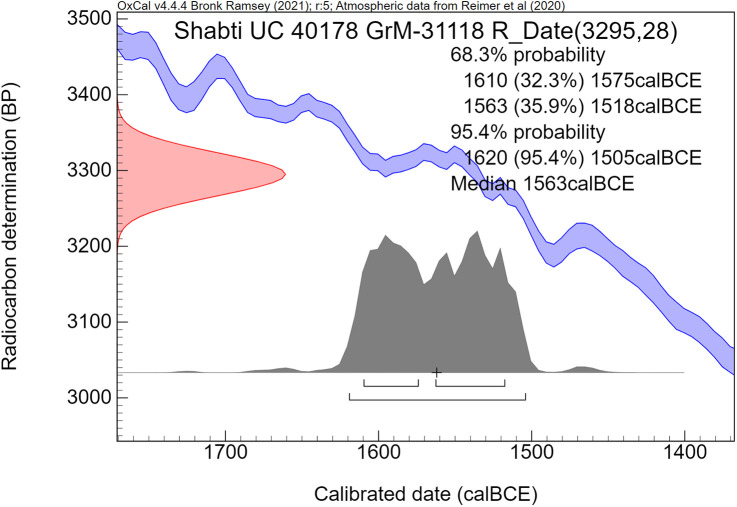
Calibrated radiocarbon age ranges for Shabti UC 40178.

The conventional uncalibrated radiocarbon date (Table 6) is 3295 ± 28 yr BP (GrM-31118). The calibrated date has, as usual, an irregular shape (Fig 13). The 68.3% probability range shows two calibrated ages: 1610–1575 cal BCE (32.3%) and 1563–1518 cal BCE (35.9%), respectively (Table 6). The first age range is firmly situated in the 17^th^ Dynasty. The second age range covers the transition from the 17^th^ to the 18^th^ Dynasty.

The historical dating by Petrie [86] of shabti UC 40178 to the 17^th^ Dynasty is confirmed by linguistic dating, as the hieroglyphic writing on this shabti of the god Sokar “*with the two diagonal strokes is the most common form used during the Second Intermediate Period*” [95,182, p. 3]. Our radiocarbon dating result also supports a date for shabti UC 40178 in the 17^th^ Dynasty.

Who was the unnamed king mentioned in the hieroglyphic text on the shabti “*an offering which the king gives*…”? Looking at the 17^th^ Dynasty kings in Table 2 and the calibrated radiocarbon ranges of shabti UC 40178, it may have been one of the three Antef kings or even earlier? The uncalibrated ^14^C date (GrM 31118, 3295 ± 28 BP) of shabti UC 40178 is about 65 radiocarbon years older than the pure straw fragment in the Ahmose brick (GrA-64347, 3230 ± 60 BP). The name of the deceased person on this shabti, Teta-sa-antef, meaning Teta son of Antef, may hint to a period in which the name Antef was popular.

### Wooden stick shabti UC 40179

**Fig 14:**
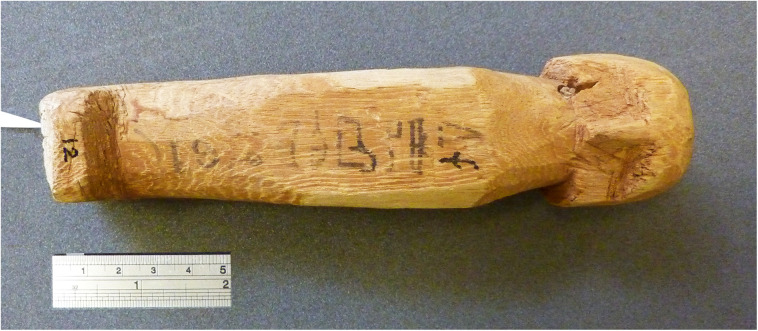
Shabti UC 40179. Photo by H.J. Bruins (2017), published with permission from the Petrie Museum of Egyptian and Sudanese Archaeology (University College London) under a CC BY license.

Shabti UC 40179, nr. 12 in Petrie’s classification, as written on its feet part (Fig 14), is also related by him to the 17^th^ Dynasty [94]. Its dimensions are 16.6 cm high, 4.1 cm wide, and 4.3 cm in diameter [95]. Compared with the 11 distinguished shabti head styles, shabti UC 40179 clearly belongs to group G, according to Whelan [95]. This type is characterized in particular by the head being wider than the neck (Fig 14), due to prominent representation of the hair mass (wig). Three columns of crude black hieroglyphs appear on the front, the right side and the back, which Whelan translated [95, p. 73]: “*An offering which the king gives to Osiris lord of Abydos so that he may give a voice offering of bread and beer for the ka of Tet(i)-sa-(intef) making his name live (by) Teti-(mes)u*.”

Whelan [95, p. 70, 73] noted that the name of the dedicator Teti-(mes)u, written in hieratic, occurs also on shabti UC 40177, which we did not investigate with radiocarbon. But most important, the name Teti-mesu is also attested on figures in the tomb complex TT15 of Tetiky in Dra Abu el-Naga (Thebes) [95,183,184]. Tetiky, spelled as Teta-Ky by the excavators [183] was the mayor of Thebes during the reign of Nebpehtire Ahmose [185–187]. Tetiky apparently continued as mayor of Thebes into the reign of Amenhotep I, the son of Ahmose. Therefore, If the dedicator Teti-mesu is the same person as the one attested on figures in tomb TT15 of Tetiky, shabti UC 40179 may be synchronous with part of the reigns of the first two kings of the 18^th^ Dynasty. The significance of this tomb has been emphasized by Christiane Ziegler [188, p. 553]: “*Located at Dra Abu’l Nagga, the tomb of Prince Tetiky (TT 15) is one of the most interesting New Kingdom tombs because of its early date — the very beginning of the Eighteenth Dynasty — and the originality of its painted decoration*”.

The tiny wood sample of shabti UC 40179, taken from its feet part, as indicated by a white arrow (Fig 14), yielded an uncalibrated radiocarbon date (Table 6) of 3224 ± 30 yr BP (GrM-12680). This result is very similar as our ^14^C date for the Ahmose mudbrick, based on the pure straw sample (GrA-64347, 3230 ± 60 yr BP). Shabti UC 40179 is only about 6 radiocarbon years younger than the former, which represents the last years (ca year 22) of the reign of Ahmose. The similar ^14^C dates of the two samples confirm their suggested historical relationship, based on the name Teti-mesu. Therefore, the ^14^C date of shabti UC 40179 gives independent support for a low chronology of the reigns of Nebpehtire Ahmose and Amenhotep I.

**Fig 15:**
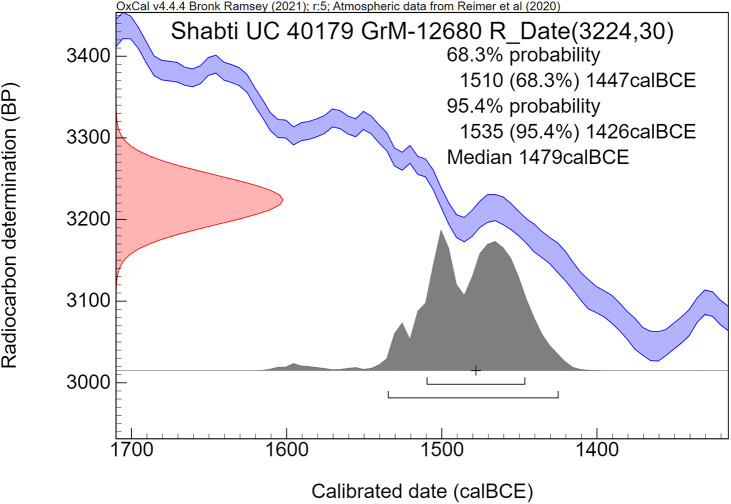
Calibrated radiocarbon age ranges for Shabti UC 40179.

The 95.4% probability calibrated age range of shabti UC 40179 (Fig 15) is 1535–1426 cal BCE. The calibration graph shows two peaks, having their highest probability centered around 1500 cal BCE and 1465 cal BCE. These calibrated radiocarbon dating results, both situated in the early 18th Dynasty, support the historical likelihood that the death of Tetiky and the construction of his tomb TT15 occurred during the reign of Amenhotep I. The two lowest historical assessments for his reign (Table 2) are 1514–1494 BCE [Hornung et al 2006} and 1498–1477 BCE [Krauss and Warburton 2009]. Hence the position of the tall peak around 1500 cal BCE (Fig 15) of the calibrated radiocarbon age range fits the above historical dating options. Our dating results support a low chronology for the first two kings of the early 18^th^ Dynasty.

### Wooden stick shabti UC 40181

**Fig 16:**
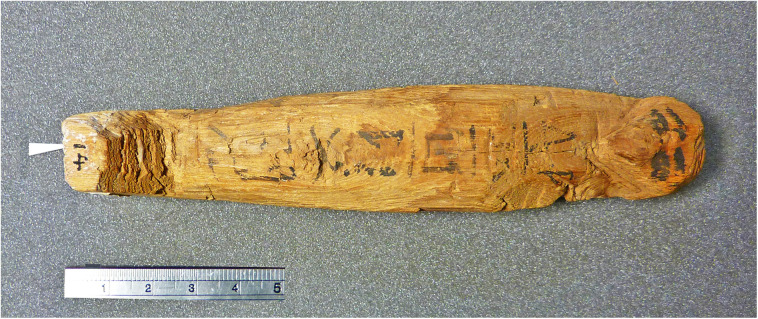
Shabti UC 40181. Photo by H.J. Bruins (2017), published with permission from the Petrie Museum of Egyptian and Sudanese Archaeology (University College London) under a CC BY license.

Shabti UC 40181, Teta-sa-antef, nr. 14 in Petrie’s classification, as visible on its feet part (Fig 16), is also assigned by him to the 17^th^ Dynasty [94]. The shabti dimensions are 13.7 cm high, 2.6 cm wide, and 2.7 cm in diameter [95]. The head style, characterized by a pointed chin (Fig 16), belongs to group B [95]. The eyes and eyebrows are painted with black ink. A column of black hieroglyphs appears only on the front side of shabti UC 40181. The translation of the text by Whelan is as follows [95, p. 76]: “*An offering which the king gives for the ka of Teti-sa-intef*.” Unfortunately, the name of the king is not mentioned.

**Fig 17:**
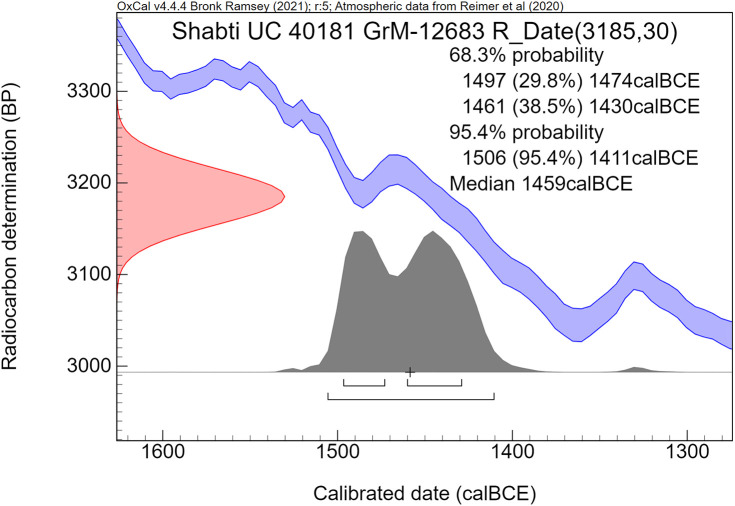
Calibrated radiocarbon age ranges for Shabti UC 40181.

Wood splinters for radiocarbon dating were taken from the bottom part of shabti UC 40181 (Fig 16, white arrow). The ^14^C date (GrM-12683, 3185 ± 30 BP) shows it to be the youngest of all six shabtis investigated (Table 6), about 45 radiocarbon years younger than the straw fragment in the mudbrick of Nebpehtire Ahmose (GrA-64347, 3230 ± 60 BP). The 68.3% probability calibrated date (Fig 17) comprises two age ranges, 1497–1474 (29.8%) and 1461–1430 (38.5%) cal BCE, which clearly belong to the early 18^th^ Dynasty. Also the 2σ (95.4%) calibrated range, 1506–1411 cal BCE, is altogether younger than the 17^th^ Dynasty, disproving Petrie’s age assessment of this shabti [94]. Considering the two 1σ calibrated peaks of highest probability (Fig 17), shabti UC 40181 might be correlated in time with Thutmose I, Thutmose II, or with Hatshepsut & Thutmose III (Table 2).

### Wooden stick shabti UC 40182.

**Fig 18:**
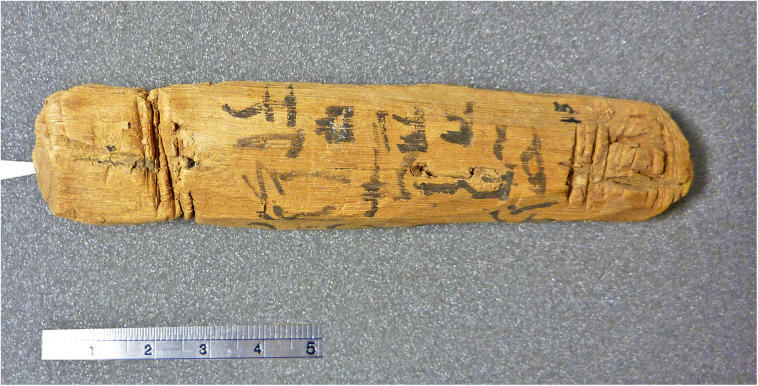
Shabti UC 40182. Photo by H.J. Bruins (2017), published with permission from the Petrie Museum of Egyptian and Sudanese Archaeology (University College London) under a CC BY license.

Shabti UC 40182, also having the name Teta-sa-antef, is nr. 15 in Petrie’s classification. The number is visible in Fig 18 on the right. Petrie also attributed this shabti to the 17^th^ Dynasty [94]. Its sizes are 11.9 cm high, 2.5 cm wide, and 2.3 cm in diameter [95]. This is the most rudimentary of all shabtis in the Petrie collection, according to Whelan [95]. It is hard to distinguish the difference between the head and feet part (Fig 18) of this crude mummiform stick shabti, as noted by Whelan [95, p. 77]: “*the head end possibly follows the direction of inscription, although the ‘foot’ end appears to have a rudimentary nose indicated*”.

The text on the shabti consists of three vertical and one horizontal column in a disorderly arrangement, using both cursive hieroglyphs and hieratic signs, translated as follows by Whelan [95, p. 77]: “*An offering which the king gives to Osiris (lord of) Djedu (lord?) so that he may give a voice offering for the ka (of) Teti-sa-intef.*”

The white arrow (Fig 18) indicates where tiny wood samples for radiocarbon dating were sampled. The ^14^C date of shabti UC 40182 is 3288 ± 30 BP (GrM-12684), being very similar as the date of shabti UC 40178 (Table 6), 3295 ± 28 BP (GrM-31118). Since the deceased person of both shabtis has the same name, Teta-sa-antef, the ^14^C dating similarity may indicate that these shabtis were made for the same individual.

**Fig 19:**
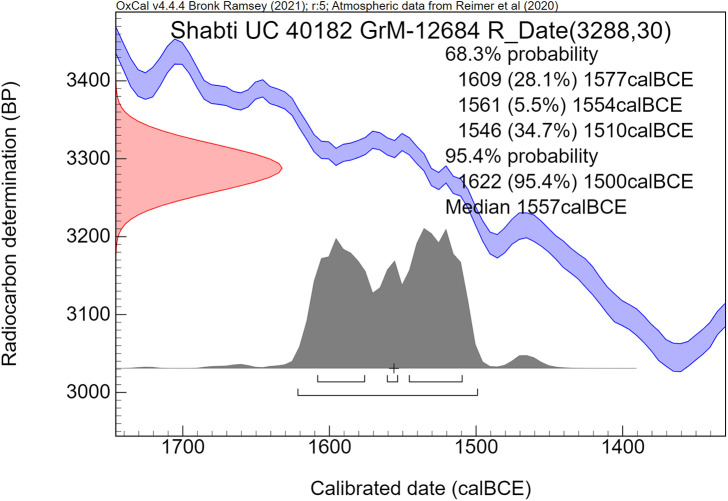
Calibrated radiocarbon age ranges for Shabti UC 40182.

The uncalibrated ^14^C date (GrM-12684, 3288 ± 30 BP) of shabti UC 40182 is about 60 radiocarbon years older than the pure straw fragment in the Ahmose brick (GrA-64347, 3230 ± 60 BP). The calibration curve is rather flat over this time trajectory (Fig 19), resulting in 68.3% probability age ranges that fit the 17^th^ Dynasty, 1609−1577 (28.1%), 1561−1554 (5.5%) cal BCE, but also overlaps with the earliest part of 18^th^ Dynasty: 1546−1510 (34.7%) cal BCE. The older time options support a 17^th^ Dynasty age, as suggested by Petrie [94].

### Wooden stick shabti UC 40184

**Fig 20:**
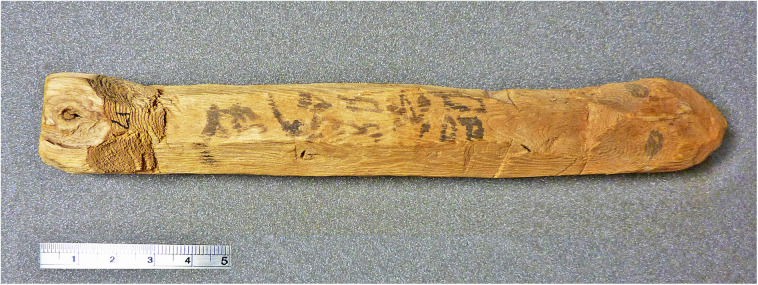
Shabti UC 40184. Photo by H.J. Bruins (2017), published with permission from the Petrie Museum of Egyptian and Sudanese Archaeology (University College London) under a CC BY license.

Shabti UC 40184 is nr. 17 in Petrie’s classification, as written on the bottom front part (Fig 20). Petrie [94] dated this shabti to the 17^th^ Dynasty. Its dimensions: 18.6 cm high, 2.6 cm wide, and 3.1 cm in diameter [95]. The head style of this shabti belongs to group D, characterized by two diagonal cuts that form the nose [95]. The eyes are indicated with black ink (Fig 20). A column of black hieroglyphs appears on the front side and another on the left side. The hieroglyphic text has been translated by Whelan [95, p. 80]: “*Teti-sa-intef made by his brother Teti-ankh*.”

**Fig 21:**
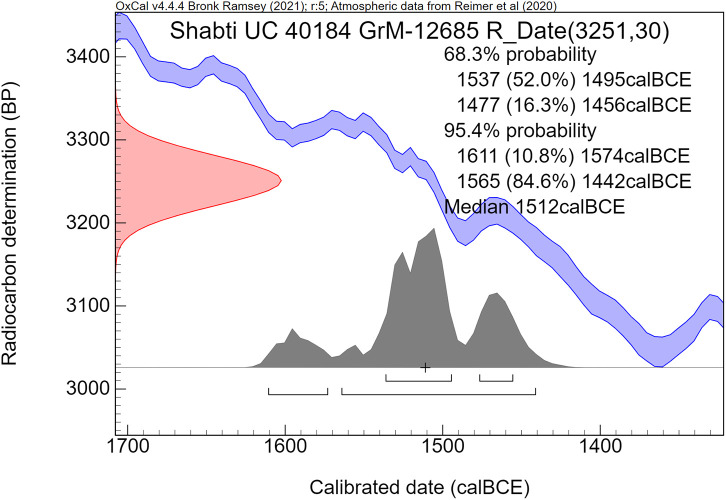
Calibrated radiocarbon age ranges for Shabti UC 40184.

Tiny wood samples for radiocarbon dating were taken from the bottom (feet) part. The uncalibrated radiocarbon date (Table 6) of shabti UC 40184 is 3251 ± 30 BP (GrM-12685). The calibrated date (Fig 21) is rather complex and ambiguous, showing three peaks. The most probable 1σ calibrated age range (52.0%) is 1537−1495 cal BCE, which could fit the reign of Nebpehtire Ahmose or Amenhotep I. A second peak has a lower probability (16.3%) age range of 1477−1456 cal BCE, partly coinciding with the reign of Hatshepsut & Thutmose III (Table 2). A third peak of lower probability (10.8%) within the 95.4% probability range, having an age range of 1611−1574 cal BCE, matches with the 17^th^ Dynasty. The median calibrated value, 1512 cal BCE, coincides with the highest peak, underlining that a date in the early 18^th^ Dynasty is most likely for shabti UC 40184.

### Wooden stick shabti UC 40196

**Fig 22:**
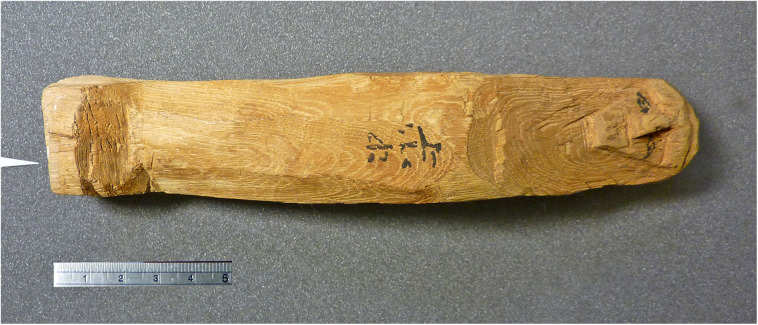
Shabti UC 40196. Photo by H.J. Bruins (2017), published with permission from the Petrie Museum of Egyptian and Sudanese Archaeology (University College London) under a CC BY license.

The only shabti in this investigation not related to the name Teta-sa-antef, or variations thereof, is shabti UC 40196, which is nr. 29 in Petrie’s classification [94], also related by him to the 17^th^ Dynasty. Its dimensions: 18.6 cm high, 3.5 cm wide, and 3.7 cm in diameter [95]. This shabti only shows the name Djehuty (Fig 22), which appears in black hieratic on the front side [95]. Its head style belongs to group H according to Whelan [95], characterized by a wedge-shaped face, a square chin, prominent nose, horizontal mouth cut, and black painted eyes below pronounced sculptured eyebrows [95]. Amongst the six shabtis investigated, UC 40196 has the most realistically proportioned face [95].

**Fig 23:**
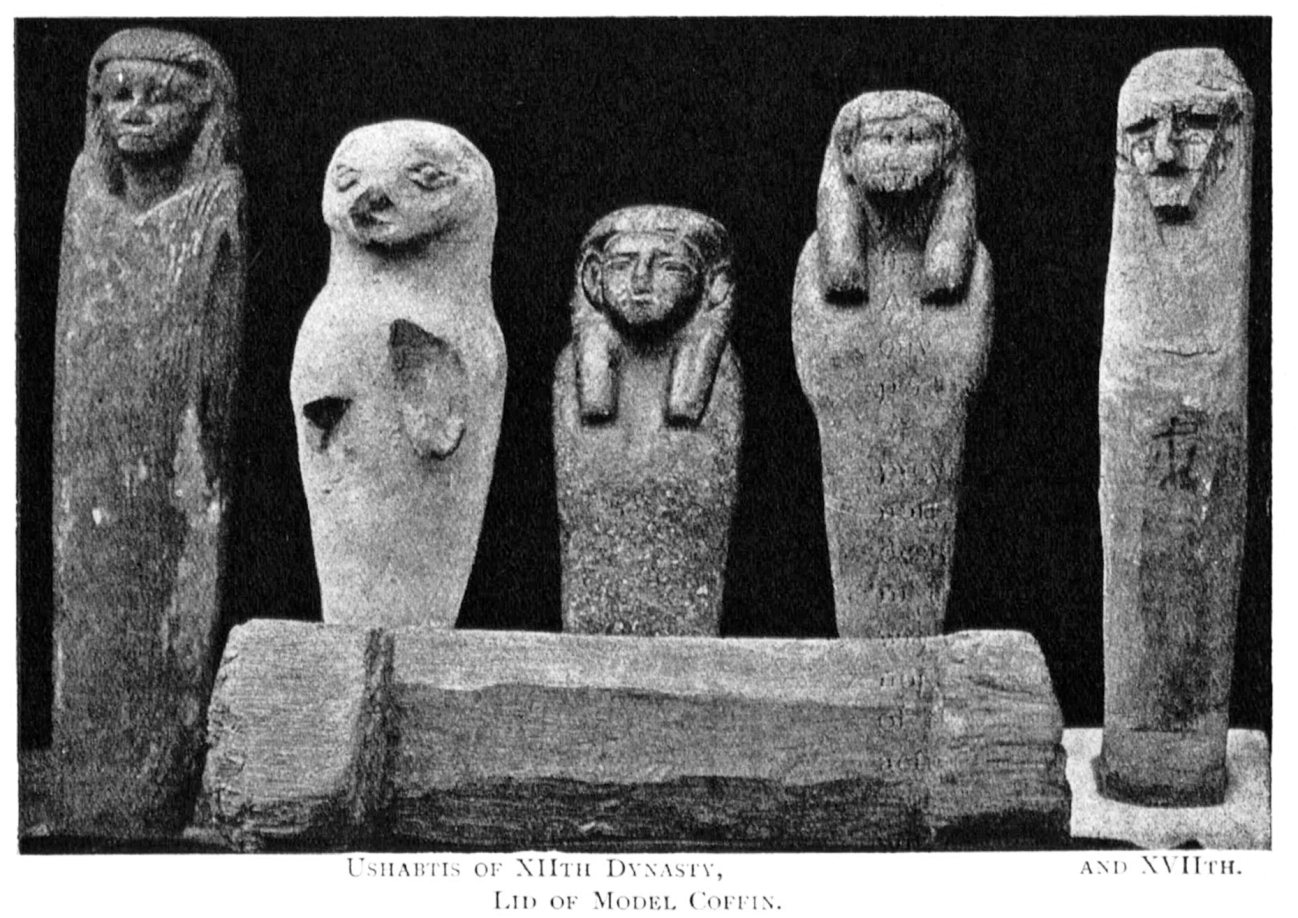
Photograph published in 1916 by Petrie, showing four 12^th^ Dynasty shabtis and one shabti attributed by him to the 17^th^ Dynasty (on the right), which can be recognized as shabti UC 40196 (Fig 22). Courtesy of the Petrie Museum of Egyptian and Sudanese Archaeology (University College London), reproduced with permission under a CC BY license.

This shabti appears also in a photograph, published by Petrie [189] in 1916 (Fig 23). It seems that by then Petrie had not yet catalogued his shabtis, as noted by Whelan [95]. Some 19 years later Petrie [94] published his main work on shabtis with a detailed catalogue, as well as illustrations of those in the Egyptian Collection at University College, London. He arranged the photographs of the shabtis according to dynasty, beginning with the 12^th^, followed by the 17^th^ and onwards to the 30^th^ Dynasty. All stick shabtis were assigned by Petrie to the 17^th^ Dynasty.

**Fig 24:**
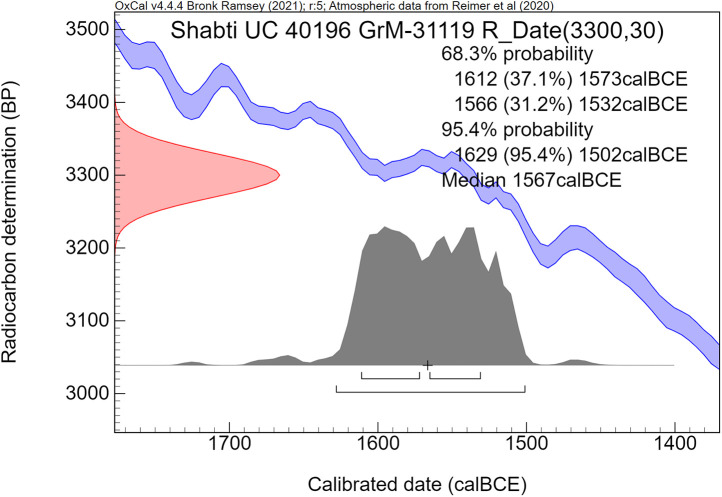
Calibrated radiocarbon age ranges for Shabti UC 40196.

Tiny splinters for ^14^C dating were taken from the bottom (feet) part, as indicated by the white arrow (Fig 22). Radiocarbon measurements yielded the oldest date, 3300 ± 30 BP (GrM-31119), of the six investigated shabtis (Table 6), which is about 70 radiocarbon years older than the straw fragment in the Ahmose brick (GrA-64347, 3230 ± 60 BP). The 68.3% probability calibrated age ranges of shabti 40196 (Fig 24) are 1612−1573 (37.1%) cal BCE and 1566−1532 (31.2%) cal BCE, supporting a date in the 17^th^ Dynasty, as suggested by Petrie. Due to the plateau in the calibration curve, there is also some overlap with the earliest part of the 18^th^ Dynasty.

### The Minoan Thera eruption vis-à-vis Egypt’s 17^th^ to early 18^th^ Dynasty: Comparing uncalibrated ^14^C dates

Following the presentation and discussion of our novel radiocarbon measurements of Egyptian museum objects of the 17^th^ to early 18^th^ Dynasty transition, we are now able to make temporal comparisons with the Minoan Thera eruption, based on the same methodology [41,190]: ^14^C time space. The uncalibrated radiocarbon dates of the Minoan Thera (Santorini) eruption, which we selected from available data in the literature, are from three different locations: (a) Akrotiri on the island of Thera, (b) an olive branch at Thera, (c) Palaikastro in Crete (Fig 1).

**Fig 25:**
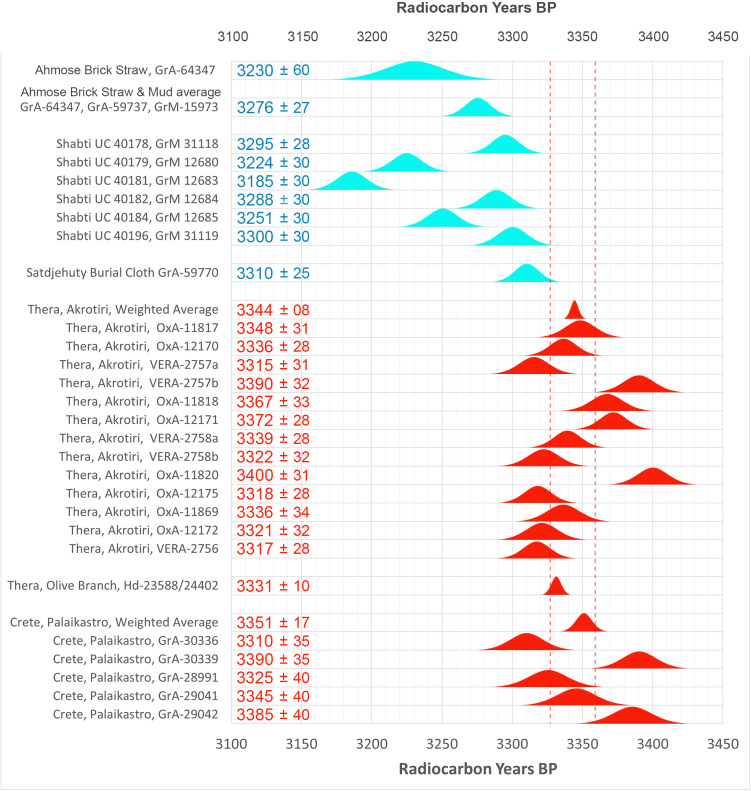
Graphic comparison of uncalibrated radiocarbon dates: Egypt’s 17^th^ to 18^th^ Dynasty transition (blue), and the Minoan Thera eruption (red). The latter dates are consistently older than the former. The two red dotted vertical lines roughly indicate the most likely ^14^C time range in which the eruption occurred, a specific geological event that took place within a certain year. On the other hand, each ^14^C date of the Egyptian museum objects represents its own temporal position within the 17^th^ to early 18^th^ Dynasty period.

Concerning Akrotiri, we selected 13 dates, having a small standard deviation of ca 30 years BP, measured on charred seeds from secure Late Minoan IA archaeological contexts in the volcanic destruction layer [57,191]. These ^14^C dates, measured in Oxford (OxA) and Vienna (VERA), are derived from four archaeological samples excavated at Akrotiri during 2000–2001: M2/76 N003 from vase A12, M7/68A N004 from basket M05, M10/23A N012 from pithos A15, M31/43 N047 from pithos A105. The 13 *individual* dates have a range from 3400 ± 31 BP (OxA-11820) to 3315 ± 31 BP (VERA-2757). The weighted average is 3344 ± 8 BP (Table 7, Fig 25). The calculation passes the Chi-square test [df = 12 T = 10.7(5% 21.0)], indicating that the 13 dates form a consistent group. The range of the individual dates and the weighted average (Table 7, Fig 25) represent a time signature of the Minoan Thera eruption in uncalibrated radiocarbon years.

**Table 7:** Comparison of uncalibrated ^14^C dates between Egypt’s 17^th^ to 18^th^ Dynasty transition (blue) and the Minoan Thera Eruption (yellow).

Sample Info	Material	^14^C Lab #	^14^C Date (yr BP)	δ^13^C (‰)
***Ahmose Mudbrick, Temple of Ahmose, Abydos, Egypt***				
British Museum, EA 32689	Pure straw fragment	GrA-64347	3230 ± 60	−12.4
British Museum, EA 32689	Mixed straw & brick fragments	GrA-59737	3290 ± 40	−23.5
British Museum, EA 32689	Mixed straw & brick fragments	GrM-15973	3285 ± 45	−24.9
*Weighted Average, Ahmose Mudbrick*			*3276 ± 27*	
***Linen Burial Cloth associated with Satdjehuty, Egypt***				
British Museum, EA 37106	Linen	GrA-59770	3310 ± 25	−25.0
***Petrie’s Wooden Stick Shabtis, Thebes, Egypt***				
Petrie Museum, UC 40178	Wood (Ficus Sycomorus)	GrM-31118	3295 ± 28	−26.9
Petrie Museum, UC 40179	Wood (Ficus Sycomorus)	GrM-12680	3224 ± 30	−26.2
Petrie Museum, UC 40181	Wood (Ficus Sycomorus)	GrM-12683	3185 ± 30	−29.3
Petrie Museum, UC 40182	Wood (Ficus Sycomorus)	GrM-12684	3288 ± 30	−26.8
Petrie Museum, UC 40184	Wood (Ficus Sycomorus)	GrM-12685	3251 ± 30	−26.6
Petrie Museum, UC 40196	Wood (Ficus Sycomorus)	GrM-31119	3300 ± 30	−28.5
***Akrotiri, Thera, Greece***				
M2/76 N003	? Lathyrus	OxA-11817	3348 ± 31	−22.9
M2/76 N003	? Lathyrus	OxA-12170	3336 ± 28	−22.9
M2/76 N003	? Lathyrus	VERA-2757a	3315 ± 31	−24.1
M2/76 N003	? Lathyrus	VERA-2757b	3390 ± 32	−21.5
M7/68A N004	Hordeum	OxA-11818	3367 ± 33	−25.8
M7/68A N004	Hordeum	OxA-12171	3372 ± 28	−25.7
M7/68A N004	Hordeum	VERA-2758a	3339 ± 28	−26.5
M7/68A N004	Hordeum	VERA-2758b	3322 ± 32	−24.7
M10/23A N012	Hordeum	OxA-11820	3400 ± 31	−25.2
M10/23A N012	Hordeum	OxA-12175	3318 ± 28	−24.7
M31/43 N047	Hordeum	OxA-11869	3336 ± 34	−22.8
M31/43 N047	Hordeum	OxA-12172	3321 ± 32	−23.1
M31/43 N047	Hordeum	VERA-2756	3317 ± 28	−21.6
*Weighted Average, Akrotiri*			*3344 ± 08*	
***Thera, Greece***				
Outer tree rings	Olive Branch	Hd-23588/24402	3331 ± 10	
***Palaikastro, Crete, Greece – Geoarchaeological Tsunami Deposits with Tephra from Thera Eruption***				
Near Building 6	Tooth lower jaw, Goat/Sheep	GrA-29042	3385 ± 40	−20.13
Near Building 6	Lower jaw, Goat/Sheep	GrA-29041	3345 ± 40	−20.14
Near Building 6	Lower jaw, Goat/Sheep	GrA-28991	3325 ± 40	−20.20
Promontary	Cattle bone	GrA-30339	3390 ± 35	−18.71
Promontary	Cattle bone	GrA-30336	3310 ± 35	−20.81
*Weighted Average, Palaikastro*			*3351 ± 17*	

An olive branch showing many tree rings was found buried in the Minoan eruption tephra at Thera, about 3.5 km south of the modern town of Phira [192,193]. The uncalibrated radiocarbon date of the outermost tree rings, 3331 ± 10 BP (Hd-23588/24402), fits very well in the range of individual radiocarbon dates for the volcanic destruction layer at Akrotiri (Table 7, Fig 25).

At the Minoan town of Palaikastro, situated in eastern Crete at a distance of some 160 km from the Thera volcano, extensive tsunami deposits were discovered along the coast, caused by the Minoan Thera eruption [8,9]. The chaotic tsunami deposits were found to contain volcanic tephra ash, Late Minoan IA ceramics, as well as animal bone fragments, which were dated by radiocarbon [8,9,194]. The individual ^14^C dates of Palaikastro range from 3390 ± 35 BP (GrA-30339) to 3310 ± 35 BP (GrA-30336), which is very similar as the range of ^14^C dates for the volcanic destruction layer at Akrotiri, Thera (Table 7, Fig 25).

This similarity in radiocarbon dates between Akrotiri, the Thera olive branch, both next to the volcano, and Palaikastro at 160 km south-east from the volcano, indicates that the former radiocarbon dates were not influenced by magmatic volcanic CO_2_ gas lacking ^14^C [194]. This would have caused older ^14^C dates at Thera, as compared to Palaikastro in Crete, not reflecting the actual time of the Minoan eruption. Hence, there does not seem to be a measurable volcanic CO_2_ reservoir effect in the vegetation growing on Thera (Santorini), as also noted by Manning [60] and Pearson et al. [68].

The weighted average of the five Palaikastro dates, 3351 ± 17 BP, passes the chi-square test [df = 4 T = 3.8(5% 9.5)]. The Palaikastro average is only marginally older than the average for the Akrotiri dates. Though both series of dates are from the same geological multifaceted catastrophe (volcanic eruption and tsunami), bones from domesticated animals (cattle, sheep, goats), found in the tsunami deposits at Palaikastro, tend to be a few years older than seeds from annual plants, found at Akrotiri in the volcanic destruction layer of the Minoan Thera eruption.

### Conclusions

#### The Ahmose mudbrick (EA 32689).

The 5 radiocarbon dating results of the Ahmose mudbrick (Table 4) underline findings from other investigations [70,97] that mud from alluvial Nile soil usually contains plant and organic components of different ages that are older than the “fresh” plant fragments added as straw at the time of mudbrick fabrication. Indeed, 4 samples of the Ahmose mudbrick, composed of mudbrick aggregates (lumps) and plant/organic remains of various sizes >0.2 mm (GrA-59737, GrM-15973, GrM-15201, GrM-14176/14177), yielded ^14^C dates older by 60–155 radiocarbon years than a single pure straw fragment (GrA-64347). The latter sample, which belongs to the largest plant fragments visible (Fig 5) at the surface of the mudbrick from the Temple of Ahmose at Abydos can be considered as “fresh” straw added at the time of production of the brick. Therefore, its radiocarbon date (GrA-64347, 3230 ± 60 BP) relates to ca year 22 of the reign of Nebpehtire Ahmose, when his Temple at Abydos was built *after* his victory over the Hyksos [111,112].

The large straw fragment (GrA-64347) is not derived from wheat or barley, because its δ^13^C value is −12.4 ‰ (Table 5), i.e., a plant with C4 photosynthesis. A microscopic investigation of a thin section made of an intact piece of the Ahmose mudbrick revealed a plant fragment with a length of only 1.4 mm (Fig 6). The epidermis layer shows small silicified cell bodies (phytoliths), ca 10 micron in size, which appear to have conical shapes, typical for sedge plants (*Cyperaceae* family) [143–146]. The papyrus (*Cyperus papyrus*) is a C4 plant belonging to this family, and is known to have similar δ^13^C values as our radiocarbon dated straw sample GrA-64347. Sedges and reeds are perennial plant families with both C3 and C4 species, growing in riverine locations along the Nile [150] where there is sufficient water available during every month. They have stems that may be chopped to provide straw. Unlike wheat and barley, sedges and reeds are available to provide straw for mudbrick production throughout the year.

The calibration graph of the ^14^C measurement of the pure straw fragment (GrA-64347, 3230 ± 60 BP) shows that the age range 1542–1427 cal BCE (Fig 7) has the highest probability (66.4%). The two tallest peaks within this age range are centered around 1500 and 1470 cal BCE. Mudbrick fabrication for the Temple of Ahmose at Abydos may be related historically to ca year 22 of Ahmose’s reign [111,112]. Low chronology historical options for year 22 of Amose (Table 2), 1502 BCE [89] and 1517 BCE [76], appear closest to these high probability peaks in the calibration graph. The two oldest historical chronology options (Table 2), 1548 BCE [129] and 1558 BCE [88] are situated outside the above calibrated age range of pure straw from the Ahmose mudbrick.

Radiocarbon dating supports the unique historical genealogical chronometric study by Bennett [113]. He calculated a ***minimum*** time interval of 315 years between year 7 of Senusert III (12^th^ Dynasty) and year 1 of Nebpehtire Ahmose. Such a ***minimal*** block of time, which includes the Second Intermediate Period, can only be accommodated by “***a high chronology for the Middle Kingdom (year 7 of Senusert III= 1872 or 1866) and a low chronology for the New Kingdom (year 1 of Ahmose = 1539)***” [113, p. 241]. The calculation by Bennett is independent of the fall of Avaris and the archaeology of Tell el-Dab’a [18,136,137]. A high chronology for the Middle Kingdom is supported by radiocarbon dating of organic materials related to Senusert III [74,162,163]. Bennett’s low chronology [113] for the beginning of the New Kingdom is supported by our investigation of the Ahmose mudbrick EA 32689, stamped with his throne name Nebpehtire (Fig 4), which provided the first ever radiocarbon measurements regarding his reign and the beginning of the 18^th^ Dynasty.

#### Linen burial cloth EA 37106, associated with Satdjehuty.

Queen Satdjehuty, who was the second wife of the 17^th^ Dynasty king Seqenenre Tao (Table 2), apparently died during the reign of Amenhotep I, the son of Nebpehtire Ahmose and his wife Queen Ahmose-Nefertari [166]. However, the radiocarbon measurements of linen burial cloth EA 37106 (Table 3), measured in duplo, yielded a date of 3310 ± 25 BP (GrA-59770). This is the oldest radiocarbon result we obtained in our investigation of 17^th^ to early 18^th^ Dynasty museum objects of ancient Egypt (Table 7, Fig 25). For comparison, the above uncalibrated ^14^C radiocarbon measurement of mummy wrapping EA 37106 is about 80 radiocarbon years older than the ^14^C date BP of the pure straw fragment (GrA-64347, 3230 ± 60 BP) in the Ahmose mudbrick.

The 1σ calibrated age (highest relative probability) of the burial cloth (Fig 10) gives two age ranges 1612–1573 (39.8%) and 1566–1538 (28.5%) cal BCE. These results, fitting the 17^th^ Dynasty, are unmistakably older than historical age assessments for the reign of Amenhotep I (Table 2). Therefore, a number of questions arise: (a) Perhaps mummy wrapping EA 37106 was a 17^th^ Dynasty linen heirloom given for Satdjehuty’s burial in the early 18^th^ Dynasty? (b) Satdjehuty died earlier than usually interpreted? (c) The association of burial cloth EA 37106 with Satdjehuty is possibly incorrect?

#### Petrie’s wooden stick shabtis from Thebes.

Sir Flinders Petrie acquired at Thebes about forty wooden stick shabtis, “roughly split and chopped”, which he all attributed to the 17^th^ Dynasty [94, p. 3]. We investigated six of these stick shabtis. The δ^13^C values we obtained range from −26.2 to −29.3 ‰ (Table 4 and 5), which indicate that the *Ficus Sycomorus* tree, widespread in Egypt, was used to produce the stick shabtis. Their ^14^C measurements (Tables 6, 7 and Fig 25) range from 3300 ± 30 BP (GrM-31119; shabti UC 40196) to 3185 ± 30 BP (GrM-12683; shabti UC 40181). All uncalibrated ^14^C dates of the six shabtis are younger than uncalibrated ^14^C dates of the Minoan Santorini eruption (Table 7, Fig 25).

The calibrated radiocarbon results show that Petrie’s age assessment [94] was partly correct. Three shabtis yielded calibrated age ranges (Table 6) situated largely in the 17^th^ Dynasty (UC 40178 Teta-sa-antef, UC 40182 Teta-sa-antef, UC 40196 Djehuty). Two stick shabtis date clearly to the early 18^th^ Dynasty (UC 40179 Tet-sa, and UC 40181 Teta-sa-antef). The latter shabti (UC 40181) may even date to the time of Hatshepsut/Thutmose III (Table 2). A detailed investigation by Whelan [95] of all 44 stick shabtis acquired by Petrie, kept in the Petrie Museum, led him to a broader age assessment, including both the 17^th^ and early 18^th^ Dynasty, which is now confirmed by our radiocarbon dates.

The name Teta-sa-antef as the deceased person occurs on four shabtis we investigated (Table 6). Our ^14^C dating results show that for shabtis UC 40178 and UC 40181 we are dealing with different Teta-sa-antef persons who died at dissimilar times. However, two other Teti-sa-intef shabtis have virtually the same ^14^C date: shabti UC 40178, 3295 ± 28 BP (GrM-31118) and shabti UC 40182, 3288 ± 30 BP (GrM-12684). This may indicate that both shabtis were made for the same individual.

The most significant result is from shabti UC 40179, on which the name Teti-(mes)u is written as dedicator. Whelan [95, p. 70, 73] noted that the same name Teti-mesu also occurs on figures [184] in the tomb complex TT15 of Tetiky, who was the mayor of Thebes during the reign of Nebpehtire Ahmose and apparently died during the reign of Amenhotep I [183–188]. The uncalibrated radiocarbon date of shabti UC 40179 (3224 ± 30 yr BP, GrM-12680) is virtually the same as the ^14^C date of the pure straw fragment of the Ahmose mudbrick (3230 ± 60 yr BP, GrA-64347). The latter date represents ca year 22 of Ahmose, i.e., near the end of his reign. The ^14^C date of shabti UC 40179 is only about 6 radiocarbon years younger than the straw of the Ahmose brick. Thus, radiocarbon dating confirms the suggested historical linkage [95, p. 70, 73] that the dedicator Teti-(mes)u may be the same person as the Teti-mesu attested on figures in tomb TT15 of Tetiky [184]. In conclusion, our calibrated ^14^C results of shabti UC 40179 and the large straw fragment in the Ahmose brick corroborate each other, while both dates support a low chronology for the first two kings of the 18^th^ Dynasty.

#### The Minoan Thera eruption is older than the late 17^th^ and early 18^th^ Dynasty.

Considering all our radiocarbon results of ancient Egyptian museum objects belonging to the 17^th^ and early 18^th^ Dynasties, it can be seen in Table 7 and Fig 25 that the uncalibrated ^14^C dates of the Minoan Thera eruption are older than the former. Both groups clearly have a different ^14^C time signature. The huge Minoan eruption predated the 18^th^ Dynasty and the reign of its first king, Nebpehtire Ahmose, as well as the late 17^th^ Dynasty. Therefore, our ^14^C investigation of Egyptian museum objects support a date for the Minoan Thera eruption within the Second Intermediate Period [60,67].

In conclusion, the reign of Nebpehtire Ahmose and the beginning of the 18^th^ Dynasty **postdate** the Minoan eruption. Assessments relating the Ahmose Tempest Stela at Karnak (Thebes) and its description of severe rainstorms to the Minoan Thera eruption can now be considered incorrect. Indeed, severe rainstorms in southern Egypt in the area of Thebes (Fig 1) are extremely rare, because the region has a hyper-arid desert climate with hardly any rainfall at all. Rare rainfall events in the Thebes region are not derived from the Mediterranean Sea (Thera), but are occasionally generated by the atmospheric Red Sea Trough, extending from the African Monsoon over equatorial Africa northward over the Red Sea region [195,196].

Finally, radiocarbon dating supports the unique historical chronology by Bennett [113], who was able, based on his genealogical studies of the governors of El-Kab, to calculate the **minimum** time distance between Senusert III (12^th^ Dynasty) and the accession year of Nebpehtire Ahmose. This interval, which includes the Second Intermediate Period, represents a significant block of time that can only be accommodated, according to Bennett’s conclusions [113], by a high chronology for the Middle Kingdom and a low chronology for the beginning of the New Kingdom. Previous radiocarbon investigations regarding king Senusert III support a **high** Middle Kingdom chronology [43,162,163]. Our radiocarbon investigation of the Ahmose mudbrick EA 32689, stamped with his throne name Nebpehtire (Fig 4), has now provided the first ever radiocarbon measurements regarding his reign, supporting, together with the ^14^C date of Shabti UC 40179, a **low** chronology for the beginning of the 18^th^ Dynasty.

### Supporting information

S1 File Inclusivity in global research. (DOCX)
